# Characterization of Ultra-Short plasma Cell-Free DNA in maternal blood and its preliminary potential as a screening marker for preeclampsia

**DOI:** 10.1186/s10020-025-01307-1

**Published:** 2025-07-12

**Authors:** Weiqiang Liu, Qin Lu, Weijie Xie, Liang Hu, Lijuan Wen, Shuxian Zeng, Jiatong Zhong, Nani Lin, Yanxiang Chen, Yimin Wang

**Affiliations:** 1 Central Laboratory (genetics lab), Longgang District Maternity & Child Healthcare Hospital of Shenzhen City (Longgang Maternity and Child Institute of Shantou University Medical College), Shenzhen, 518172 China; 2 Department of Applied and Translational Medicine, GeneMind Biosciences Co., Ltd, Shenzhen, 518000 China; 3 Longgang District Key Laboratory for Birth Defects Prevention, Shenzhen, 518172 China; 4 Department of Laboratory Medicine, Peking University Shenzhen Hospital, Shenzhen, 518000 China; 5 College of Pharmacy, Xiangnan University, Chenzhou, 423000 China

**Keywords:** Ultra-short cfDNA, Pregnancy, Preeclampsia, Early diagnosis

## Abstract

**Background:**

Cell-free DNA (cfDNA) in maternal blood is the foundation for non-invasive prenatal screening (NIPS). Recently, ultra-short cfDNA fragments, ranging from 30 to 70 base pairs (bp), have been identified and show promise in cancer diagnostics. However, the characteristics of ultra-short cfDNA in maternal blood during pregnancy remain unexplored. This study aims to characterize these fragments in pregnancy and evaluate their potential as biomarkers for preeclampsia.

**Methods:**

Ultra-short cfDNA was isolated from the plasma of pregnant and non-pregnant women, and next-generation sequencing (NGS) was constructed. We performed deep sequencing to profile ultra-short cfDNA during pregnancy, including a cohort of women with preeclampsia, to identify distinct ultra-short peaks. These features were used to build a diagnostic model, which was validated in a separate testing cohort.

**Results:**

Sequencing data revealed that ultra-short cfDNA in maternal blood primarily originates from accessible open chromatin regions in blood and placental cells. A significant proportion of these fragments exhibited potential G-quadruplex (G4) motifs on the antisense strand. Significant differences in ultra-short cfDNA features were observed between women with preeclampsia and healthy controls. Based on these features, the diagnostic model achieved an area under the curve (AUC) of 0.90 in the training cohort and 0.86 in the test cohort.

**Conclusions:**

This study comprehensively characterizes ultra-short cfDNA in maternal blood and suggests its preliminary potential as a diagnostic marker for early preeclampsia detection.

**Trial registration:**

Retrospectively registered.

**Supplementary Information:**

The online version contains supplementary material available at 10.1186/s10020-025-01307-1.

## Background

Cell-free DNA (cfDNA) is released into circulation after cell death or active secretion and retains the genetic and epigenetic signatures of its source (Ivanov et al. 2015). The fragmentation of cfDNA reflects the nucleosomal footprint, which provides insight into the tissue of origin (Snyder et al. 2016). In healthy individuals, the typical size of cfDNA is approximately 166 base pairs (bp), corresponding to the nucleosomal units (Lo et al. 2010). During pregnancy, while the modal size of cfDNA remains around 166 bp (Lo et al. 2010), cell-free fetal DNA(cffDNA) typically measures 143 bp, reflecting fetal origin (Sun et al. 2018; Liang et al. 2018; Qiao et al. 2019a, b).

Recent studies have identified cfDNA fragments shorter than the nucleosomal sizes (Snyder et al. 2016; Burnham et al. 2016), with fragments ranging from 50 to 120 bp overlapping transcription factor binding sites (TFBSs) (Snyder et al. 2016), potentially protected by transcription factors (TFs). Advances in cfDNA purification and library preparation techniques have revealed ultra-short cfDNA fragments, particularly useful for detecting fragmented tumor-derived DNA in cancer diagnostics (Mouliere et al. 2011; Hisano et al. 2021). Interestingly, these fragments are less abundant in cancer patients than expected, indicating a potential diagnostic role in cancer classification(Hudecova et al. 2022).

The ultra-short cfDNA fragments, mapped to the antisense strands of genomic regions, could adopt a G-quadruplex (G4) DNA secondary structure (Hisano et al. 2021; Hudecova et al. 2022). However, the diagnostic potential of ultra-short cfDNA for cancer classification has been underscored. In this study, we demonstrate the presence of ultra-short cfDNA in the plasma of pregnant women. We further show that these fragments exhibit significant differences in women with preeclampsia compared to those with normal pregnancies, suggesting their preliminary potential as molecular markers for predicting preeclampsia.

## Methods

### Sample collection

Blood samples were collected from non-pregnant and pregnant women at different gestational weeks, with the approval of the Medical Ethics Committee of Shenzhen Longgang District Maternal and Child Health Hospital (No. KYXM-2023-036). Preeclampsia patients and healthy controls were retrospectively enrolled based on gestational age at plasma collection and follow-up data from Longgang District Maternity & Child Healthcare Hospital. Informed consent was obtained from all participants. Blood was drawn into EDTA-containing tubes, and plasma was separated within six hours by two rounds of centrifugation (first at 1600 g for 10 min, followed by a second centrifugation at 14,000 rpm for 10 min). To prevent the effects of freeze-thaw cycles, all plasma samples were aliquoted into 1 mL centrifuge tubes and stored separately at −80℃ until DNA extraction.

### CfDNA purification and library preparation

cfDNA was isolated using the Solid Phase Reversible Immobilization (SPRI) method (Cheng et al. 2022). Briefly, 20µL of 20 mg/mL protease K (Invitrogen, 25530049) and 11µL of 20% SDS (Invitrogen, 28364) were added to 200 µL plasma. The mixture was incubated at 60℃ for 30 min, then cooled to room temperature. Subsequently, 108µL SPRIselect beads (Beckman Coulter, B23319) and 600 µL isopropyl alcohol were added, and the mixture was incubated at room temperature for 10 min. The supernatant was removed once the solution became clear on a magnetic rack. The resulting pellet was resuspended in 500 µL TE buffer and incubated for 5 min, followed by further magnetic separation. The supernatant was then transferred to a new tube, and 250 µL phenol, 240 µL chloroform, and 10µL isoamyl alcohol (SOLARBIO, P1012) were added. The mixture was vortexed for 15 s and centrifuged at 19,000×g for 5 min, repeating this step twice.

Approximately 500 µL of the aqueous phase was transferred to a new tube, mixed with 270 µL SPRIselect beads and 1500 µL isopropyl alcohol, then incubated at room temperature for 10 min. The supernatant was removed once the solution was clear on the magnetic rack. The pellet was washed twice with 3 mL 85% (v/v) ethanol, air-dried for 10 min, and then resuspended in 32 µL of nuclease-free water. After magnetic separation, 30 µL of supernatant was transferred to a new tube containing 1µL glycogen (Thermo, R0561), 44 µL 1TE buffer, 25 µL 3 M sodium acetate (Invitrogen, AM9740), and 250 µL 100% ethanol. The mixture was stored at −80 °C overnight.

The tube was centrifuged at 19,000×g for 15 min the following day, and the supernatant was removed. The precipitate was washed twice with 200 µL of 80% ethanol, air-dired, and resuspended in 30 µL of water. Next, 90 µL of AMPure XP beads and 90µL of 100% isopropanol were added, and the mixture was incubated for 10 min. The tube was placed on a magnetic rack for 5 min, the supernatant was discarded, and the beads were washed twice with 200 µL of 80% ethanol. After air drying, the pellet was resuspended in 40 µL TE buffer, incubated for 5 min, and then placed on the magnetic separation until the solution was clear. The purified cfDNA was stored at −20 °C until further use.

DNA libraries were prepared using the VAHTS ssDNA Library Prep Kit for Illumina (Vazyme, ND620). Approximately 20 µL of extracted cfDNA was used as input. During all bead clean-up steps, the bead concertation was increased to 2× to retain low-molecular-weight nucleic acids. The index reaction PCR was performed for 13 cycles. Library quantity and quality were evaluated using the Qubit dsDNA HS Assay Kit (ThermoFisher, Q32854) and LabChip^®^ DNA Assays (CLS760673, CLS760673).

### Sequencing

Small-scale sequencing was performed using Genemind Genolab (GeneLab M, Genemind, China) in paired-end mode (2 × 75 cycles). For large-scale sequencing, paired-end sequencing was performed using the Genemind Surfseq platform (SURFSeq 5000, Genemind, China) with the same parameters. The indexed reads were demultiplexed for subsequent bioinformatic analysis.

### Bioinformatics analysis

Sequenced reads were filtered, and additional nucleobases from the single-strand library preparation were trimmed using fastp v0.21.0 software in default parameters (Chen et al. 2018). For quality control, reads with a Phred quality score ≥ Q15 were retained, with up to 40% of bases allowed to be unqualified. Reads containing more than five N bases were discarded, and those shorter than 15 nucleotides were removed. The remaining reads were mapped to the reference human genome (GRCh37/hg19) using bowtie2 v2.4.2 (Chen et al. 2018). Duplicates were removed using the MarkDuplicates module from the Picard v2.27.5 (McKenna et al. 2010) tool suite. Basic statistics on BAM files, such as read counts, quality distributions, coverage depth, and fragment length, were generated using SAMtools v1.15.1 (Li et al. 2009). Unique reads were separated by fragment size using SAMtools, with fragments between 30 and 70 nt classified as ultrashort. The expression profiles of human tissue and cells were downloaded from the Human Protein Atlas (https://www.proteinatlas.org/humanproteome/single+cell/single+cell+type).

Fetal fraction estimation was based on the Y chromosome ratio. Alignments to both strands of the reference genome were subjected to peak calling using MACS2 v2.2.91 (Zhang et al. 2008), and strand-specific BAM files were converted to BigWig format using the bamCoverage module of the deepTools v3.5.2(Ramírez et al. 2016) tool suite. Coverage of the mapped cfDNA fragments around human genes, known genomic regions, and chromatin-accessible or methylated regions from ChIP-Atlas (Zou et al. 2022) was visualized using deepTools. Peaks were annotated and counted using HOMER v4.11 (Heinz et al. 2010), and overlapping peaks were analyzed using Venn diagrams with the ChIPPeakAnno R package v3.38.1 (Zhu et al. 2010).

The peaks of the G4 seq were downloaded from GEO by the ID of GSE63874, and G4 motifs were predicted by fastaRegexFinder (fastaRegexFinder from https://github.com/dariober/bioinformatics-cafe/blob/master/fastaRegexFinder/fastaRegexFinder.py?raw=true). Ultra-short peak sequences were extracted using the BEDTools v2.30.0 (Quinlan and Hall 2010). Functional enrichment of the peaks was conducted using RE-GOA(Lu et al. 2022). The base composition of each DNA sequence was calculated using fx2tab from SeqKit v0.13.2 (Shen et al. 2016) and visualized using ggplot2 R package v 3.5.1.

Heatmaps were generated using pheatmap R package v1.0.12. The relative coverage of peaks was determined as the median of the peaks, and differentially enriched peaks were identified based on a fold change > 2 and an FDR < 0.05. Differentially abundant peaks were identified through a permutation test using an in-house Python script. The fetal fraction for different fragment sizes was calculated using the Y chromosome ratio. Differentially enriched peaks were selected using the R package Boruta v 8.0.0, and model training was performed using the R package Caret v6.0-94. AUC curves were plotted using the R package pROC v1.18.5. The detailed code used in the analysis is available in the supplementary data.

## Results

### Ultra-short fragments in maternal blood

The total amount of cfDNA extracted from each sample ranged from 2 to 7 ng. Ultra-short cfDNA fragments, defined as being shorter than 50 bp, were successfully extracted from maternal plasma. We designed and performed a replicated validation experiment to investigate the reproducibility of this limited amount of starting material. 10 mL of peripheral blood from two healthy pregnant volunteers were collected, and 5 mL of plasma was separated from each sample and further divided into 1 mL replicates. Our replicated experiments firmly establish that the limited starting material can be successfully used for ultra-short fragment analysis (data not shown).

**Fig. 1:**
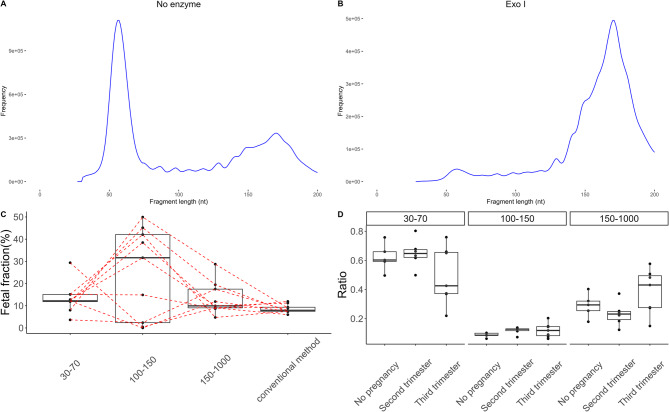
The single-strand nature of ultra-short cfDNA in pregnancy **A** Successful recovery of ultra-short cfDNA, with the insert size distribution shown; **B** The effects of nuclease treatments on a model nucleic acid mixture and cfDNA; **C** Boxplot of the fetal fraction calculated by the Y chromosome ratio over different fragment sizes, ratios were represented as a percentage of fetal cfDNA among total maternal cfDNA, and the dashed red line linked the same sample; **D** Boxplot of the ratio of fragment of different lengths in cfDNA at different stages of pregnancy, ratios were represented as decimals

Fragments of 50nt were abundant across all subjects (Fig. 1A). These short fragments resisted ribonuclease I treatment but were sensitive to DNase I, confirming their identity as DNA (Fig. 1B). Furthermore, they were susceptible to degradation by ssDNA-specific exonuclease, suggesting that they are predominantly in a single-stranded form (Fig. 1B). As the fetal fraction is a critical component for noninvasive prenatal screening and related research, it is important to understand the relationship between the cfDNA with different fragment lengths and fetal fraction. In this study, the fetal fraction, based on the Y chromosome ratio, did not show a significant difference between 30 and 70 bp and 150–1000 bp fragments, while the 100–150 bp fragments had the highest fetal fraction (Fig. 1C). Importantly, there was no significant difference in the proportion of ultra-short fragments between early and late pregnancy (Fig. 1D).

### Enrichment of ultra-short fragments in the regulatory regions

**Fig. 2:**
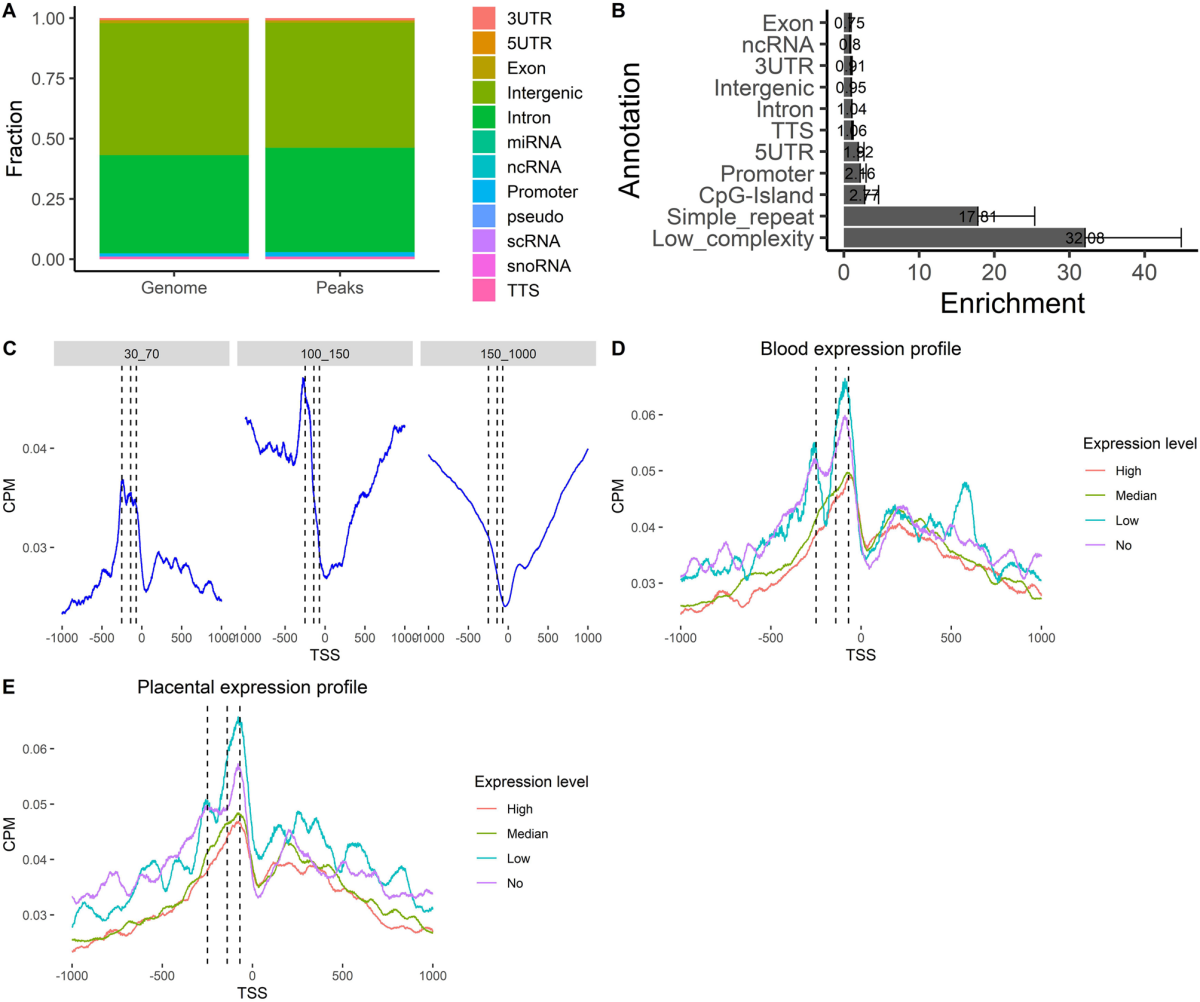
Enrichment of ultra-short cfDNA in regulatory regions of genes **A** Stacked bar plot showing the distribution of ultra-short peaks in regulatory regions; **B** Bar chart showing the enrichment of ultra-short fragments in these regulatory regions; **C** Relative coverage near the TSS evaluated over different fragment size ranges; **D** Relative coverage of ultra-short fragment near the TSS over different levels of blood expression file. **E** Relative coverage of ultra-short fragment near the TSS over different levels of placental expression file. In Figures (**C**-**E**), the x-axis represents the genomic position relative to the TSS. “0” denotes the TSS itself, “−500” indicates a position 500 base pairs upstream of the TSS, while “+500” indicates a position 500 base pairs downstream of the TSS

We sequenced 5 libraries from maternal plasma and 5 from non-pregnant women to compare the distribution of ultra-short fragments. Most ultra-short fragments originated from the nuclear genome, with only minor contributions from mitochondrial or microbial sources (Figure S1). The fragmentation patterns in both maternal and non-pregnant plasma were similar (Figure S2). The distributions of ultra-short fragments across genomic regions were evaluated, and peaks corresponding to these fragments were observed across all genomic features. Notably, ultra-short fragments were enriched in promoters, 5’ UTR, and CpG island, with the most pronounced enrichment observed in simple repeat and low complexity regions (Fig. 2A, B). In contrast to longer fragments, ultra-short fragments exhibited a sharp increase in coverage immediately upstream of the transcription start sites (TSS), with three distinct peaks at 70, 160, and 250 bp upstream, while coverage near the TSS itself was lower (Fig. 2C).

A pattern emerged when genes were categorized based on expression levels in blood or placental cells (from the HPA database): lower coverage upstream of the TSS was seen for highly expressed genes, while higher coverage was found for genes with low expression. In contrast to long fragments, the coverage of short fragments in genes with low or medium expression showed trends similar to those with no expression (Fig. 2D, E). The genomic distribution patterns of ultra-short fragments were consistent between maternal plasma and non-pregnant females (Figure S3).

**Fig. 3:**
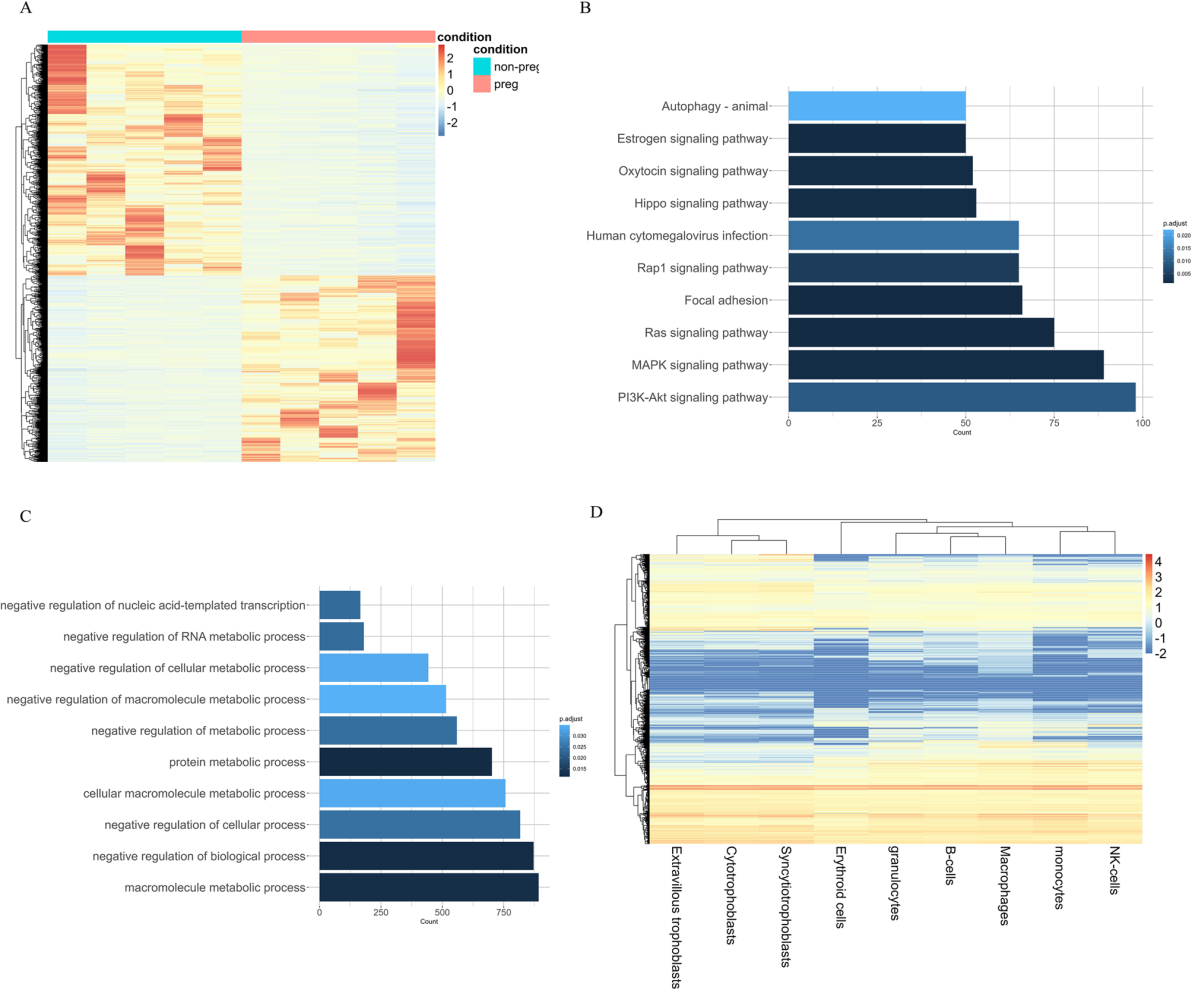
Characteristics of ultra-short fragments in pregnancy **A** Distribution of differentially abundant peaks between non-pregnant individuals and pregnant women; **B** KEGG enrichment analysis of genes associated with differentially abundant peaks; **C** GO enrichment analysis of these genes; **D** Relative expression levels of genes associated with differentially abundant peaks, retrieved from the Human Protein Atlas (HPA) database

Analysis of terminal nucleotides revealed a significant enrichment of guanine (G), with G nucleotides accounting for nearly half of all terminal bases (Figure S4). The predominant motifs were GGGG, GGGA, and GGAG (Figure S5). The differentially abundant peaks (Table S1) were associated with signaling pathways and molecular processes relevant to development (Fig. 3A), many of which exhibited high expression in blood and placental cells (Fig. 3D).

### Ultra-short fragments in accessible chromatin regions

**Fig. 4:**
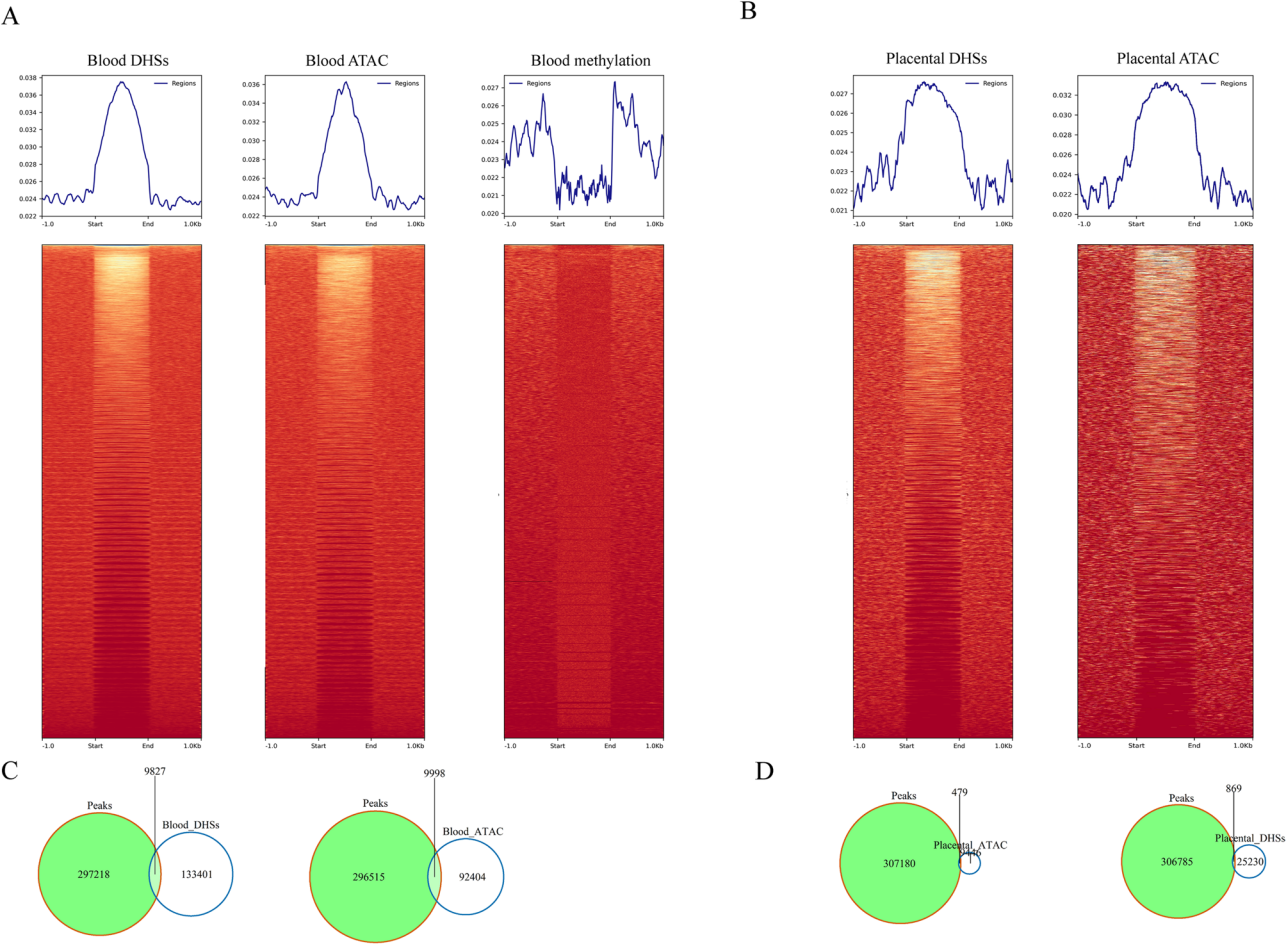
Colocalization of ultra-short cfDNA on accessible chromatin regions **A** Relative coverage of ultra-short cfDNA at blood DHSs, ATAC peaks and methylation sites. **B** Relative coverage at placental DHSs and ATAC peaks. **C**-**D** Venn diagram illustrating the overlap of ultra-short cfDNA peaks with DHSs, and ATAC-seq peaks of blood and placenta respectively

We examined the coverage of ultra-short fragments at DNase I hypersensitive sites (DHSs) and ATAC-seq peaks indicative of actively transcribed genes. Increased coverage of the ultra-short fragments was observed at DHSs and ATAC-seq sites, while decreased coverage of ultra-short fragments was observed at the boundaries of methylated regions (Fig. 4A and B, S6). In contrast, plasma from non-pregnant women did not show increased coverage at placental sites (Figure S7). Notably, the coverage at blood sites was significantly different compared to that at placental sites. Colocalization analysis revealed a significant overlap of ultra-short fragments with DHSs and ATAC-seq peaks, particularly in blood cells (Fig. 4C and D). However, these peaks accounted for only 3.3% of all ultra-short peaks. We further assessed the colocalization of these peaks with transcription factor markers in blood (Xu et al. 2022) and placental cells (Kim et al. 2023), finding that approximately 1% of all ultra-short peaks overlapped with these markers (Figure S8).

### Ultra-short fragments and G4 motifs

**Fig. 5:**
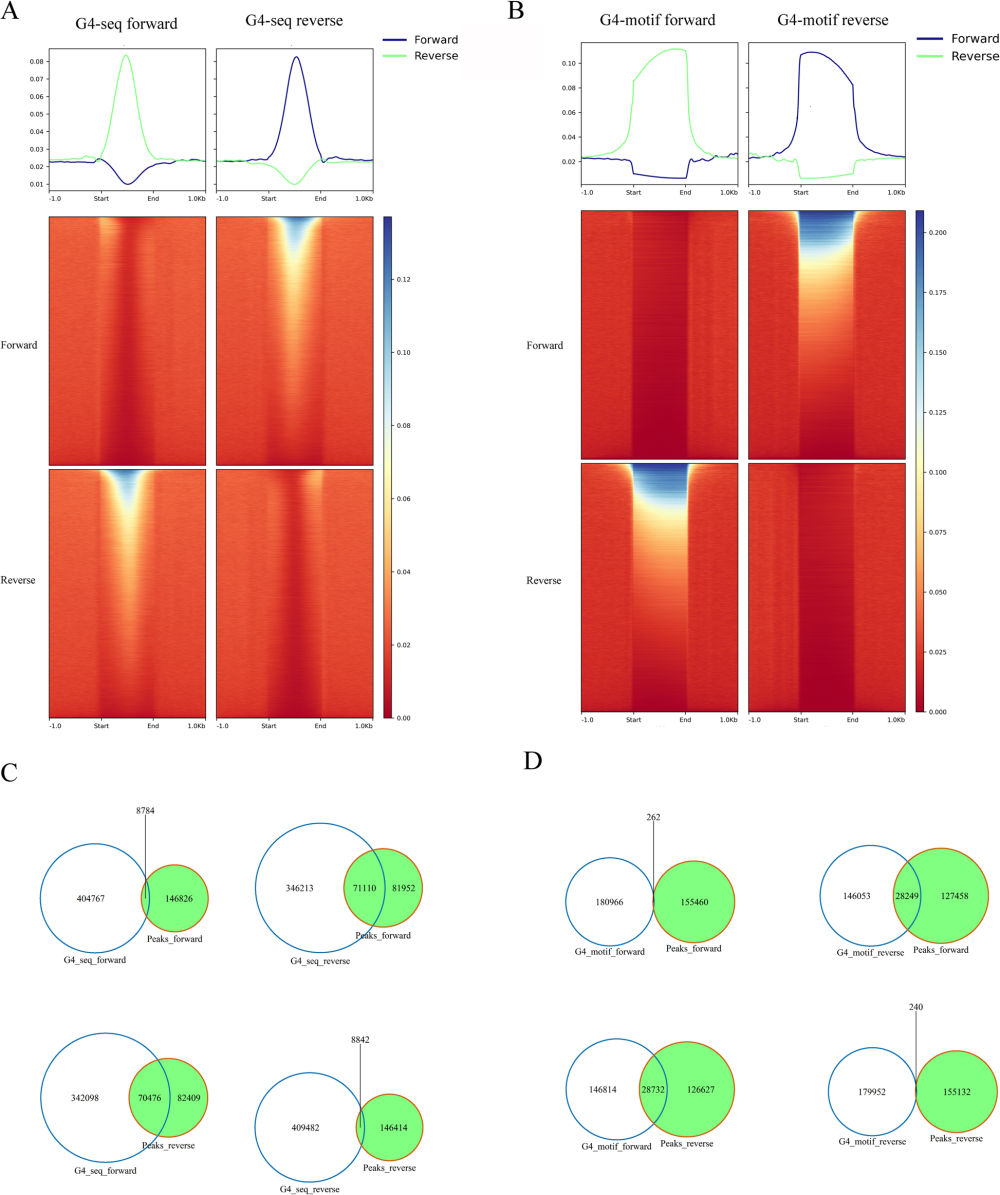
Enrichment of ultra-short fragments on complementary strands of G4 motifs. **A**-**B** Relative coverage at observed and predicted G4 sequences by strand; **C**-**D** Venn diagram showing the overlap of ultra-short cfDNA peaks and observed G4 peaks or predicted G4 motif by strand

As previously reported, C-rich reads were identified within ultra-short peaks (Figure S9), with increased relative coverage observed in potential G4 formation regions (Fig. 5A, B). Colocalization of ultra-short peaks with antisense G4 structure was identified (Fig. 5C and D), with approximately 30% of the total peaks located on the 5′-side of the ultra-short fragments. This suggested that a significant proportion of ultra-short cfDNA may originate from G4 secondary structures.

### Characteristics of ultra-short fragments in preeclampsia

**Fig. 6:**
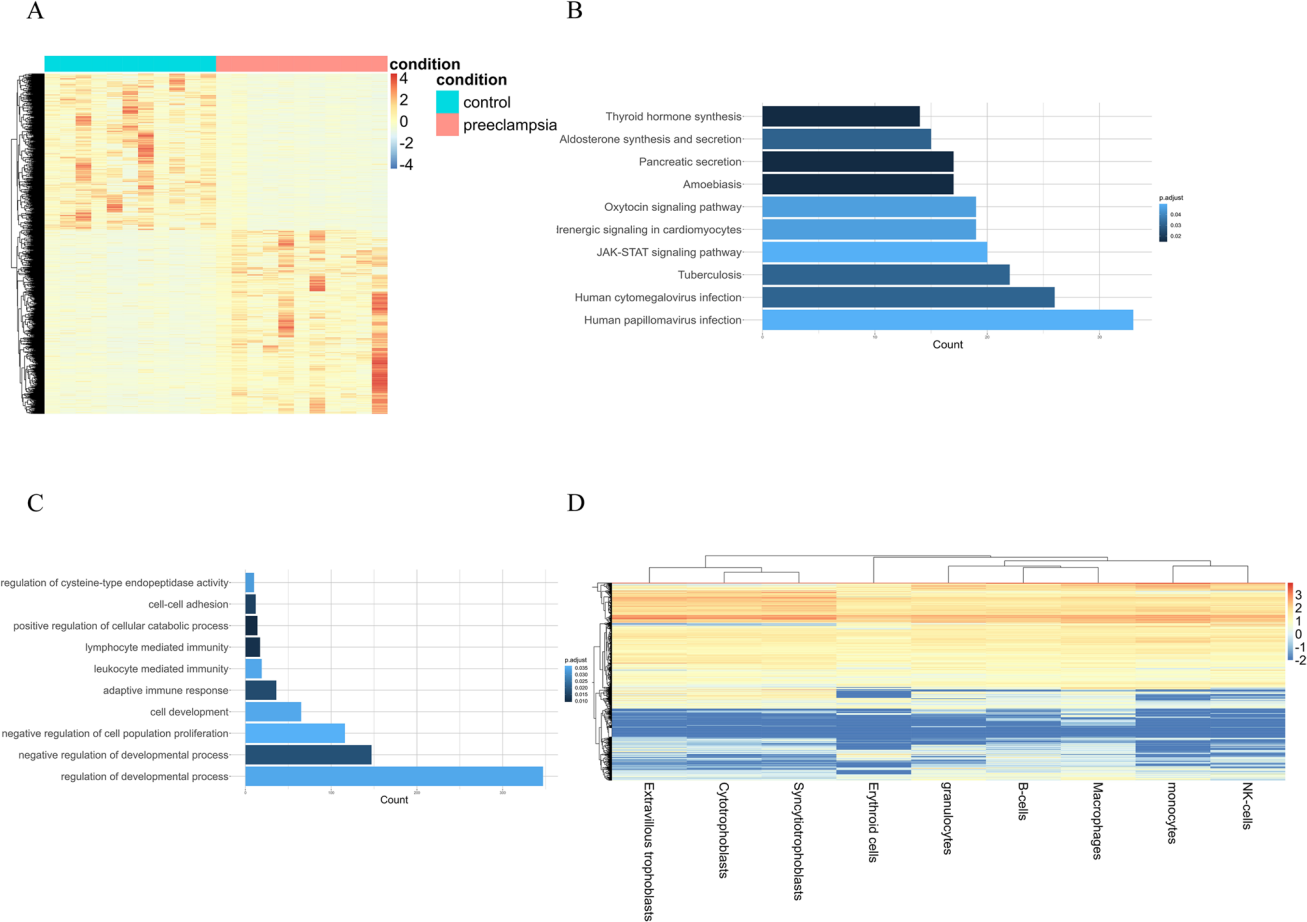
Characteristics of ultra-short fragments in preeclampsia **A** Distribution of differentially abundant peaks between healthy controls and preeclamptic patients; **B** KEGG enrichment analysis of genes associated with these differentially abundant peaks; **C** GO enrichment analysis of these genes; **D** Relative expression levels of genes associated with differentially abundant peaks, retrieved from the HPA database

Given the enrichment of ultra-short fragments in accessible chromatin regions and their association with regulatory G4 motifs, we investigated whether these fragments reflect differences in the pathological processes of preeclampsia. Preeclampsia was diagnosed as the new onset of hypertension and proteinuria, or new onset of hypertension with significant end-organ dysfunction, with or without proteinuria in previously normotensive patients. The workflow for feature selection and model building is shown in Figure S10. Deep sequencing data from plasma samples of 11 preeclampsia patients and 11 healthy controls identified over 3,000 differentially abundant peaks (Fig. 6A, Table S2). Functional enrichment analysis of genes containing ultra-short peaks at regulatory elements revealed associations with signaling pathways involved in immunity and development (Fig. 6B and C), with most of the associated genes showing high expression levels in blood and placental cells (Fig. 6D).

### Diagnostic potential of ultra-short fragments for preeclampsia

Based on the high-depth sequencing results, we performed low-pass sequencing on plasma samples collected during the second trimester from patients with preeclampsia and healthy individuals (Table S3). Four samples were excluded due to insufficient clean reads (fewer than 60 M). There was no significant difference in gestational age at blood collection, age, body mass index (BMI), and reads count between the groups, although preeclampsia patients had earlier delivery dates (Table 1). The counts per million (CPM) of the previously identified differentially abundant peaks were calculated and selected using the Boruta algorithm in a training set (56 controls vs. 48 cases) (Table S4).

**Table 1:** Summary of the enrolled pregnant women

	Control	Preeclampsia	*P* value
Number	91	79	-
Gestational age at sampling(weeks)	16.90 ± 3.05(12.86,31.71)	16.74 ± 2.60(12.71,27.14)	0.96
Age(years)	30.21 ± 4.20(19.00,41.00)	30.69 ± 4.57(20.00,41.00)	0.60
Weight	53.13 ± 9.56(39.80,90.00)	55.06 ± 9.22(37.00,90.00)	0.71
Height	157.57 ± 4.70(147.00,167.00)	157.39 ± 6.38(138.50,170.00)	0.21
BMI(kg/m^2^)	21.45 ± 3.70(16.94,35.16)	22.42 ± 3.61(16.94,36.98)	0.16
Fetal gender			
Male	49	35	0.46
Female	42	44	
FGR	0	9	0.003
Delivery time	39.17 ± 1.33(37.14,41.14)	37.06 ± 2.89(28.29,40.86)	< 0.01

*BMI* Body mass index, *FGR* Fetal growth restriction

**Fig. 7:**
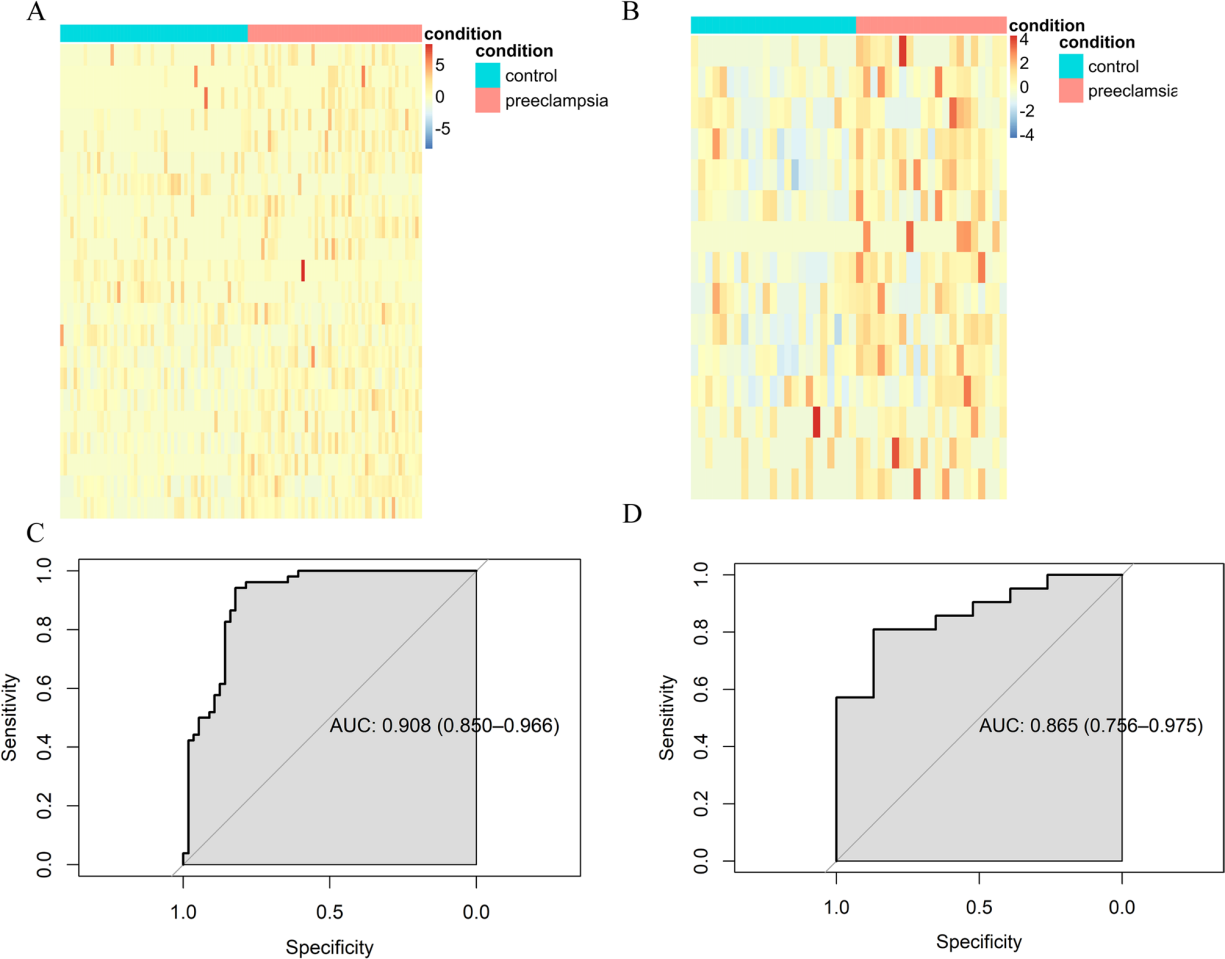
Diagnostic potential of ultra-short fragments for preeclampsia **A** Heatmap of selected region coverage in low-depth data for the training cohort; **B** Heatmap of selected region coverage in low-depth data for the test cohort; **C** AUC curve of the SVM model for the training cohort; **D** AUC curve of the SVM model for the testing cohort

An SVM model was trained and tested on 22 features, yielding an AUC of 0.90 on the training set and 0.86 on the test set (24 controls vs. 20 cases) (Fig. 7). These results demonstrate the potential of ultra-short fragments as diagnostic markers for preeclampsia in early pregnancy. In contrast, more than 500 different abundant coverage regions near the TSS for fragments longer than 150 bp were identified in the discovery cohort, though these features exhibited poor performance on the test set (Figure S11).

## Discussion

In the present study, we identified and characterized ultra-short fragments of approximately 50 bp in maternal blood during pregnancy using optimized methods for cfDNA purification and library construction. These fragments were enriched in regulatory chromatin regions of both blood and placenta regions, suggesting an association with gene expression profiles. Notably, these ultra-short fragments may potentially serve as diagnostic markers for pregnancy complications, such as preeclampsia, highlighting their potential role in regulatory mechanisms associated with pregnancy.

Our findings demonstrated that the majority of ultra-short cfDNA exists in a single-stranded form. However, the biological origin of these fragments and the mechanisms that control their conversion from double-stranded to single-stranded forms remain unclear. It is known that DNA fragmentation during cell apoptosis occurs through a stepwise process involving nucleases such as DFFB and DNASE1L3, which preferentially cleave specific nucleotides (Zhu et al. 2023; Widlak and Garrard 2005). After release into plasma, serum DNASE1L3 further fragments the extracellular DNA, resulting in a predominance of C-terminal fragments, which can then be further degraded by DNASE1 into short T-terminal fragments(Han et al. 2020). The observed enrichment of G nucleotides at the ends of ultra-short cfDNA suggests a distinct fragmentation profile, referred to as the founder profile F-profile V (Zhou et al. 2023), indicating the potential involvement of additional cleavage pathways in generating these fragments. However, the F-profile V was not directly associated with the nucleases previously implicated in cfDNA fragmentation (Han et al. 2020), suggesting that some other cleavage pathways may contribute to the formation of ultra-short cfDNA. Consequently, the stepwise cleavage by DFFB and DNASE1L3 is likely to account for only a fraction of these fragments.

We observed that the fetal fraction in ultra-short fragments was comparable to that in fragments ranging from 150 to 1000 bp, with the highest fetal fraction occurring in the 100 to 150 bp range. This is consistent with previous studies, which have been shown that fetal cfDNA is predominantly around 143 bp (Sun et al. 2018). While shorter cfDNA fragments (107–145 bp) have been associated with an increased fetal fraction (Liang et al. 2018; Qiao et al. 2019a, b), ultra-short fragments were previously more challenging to extract using conventional methods(Vong et al. 2017). Our optimized extraction protocols enabled the successfully identified ultra-short fragments derived from maternal blood and placenta, with no significant preference for placental origin. Interestingly, the fetal fraction of these ultra-short fragments increased with gestational age, mirroring trends observed for normal-sized fragments (Hui and Bianchi 2020).

Similar to previous studies (Hudecova et al. 2022), we observed three prominent peaks in coverage at positions 70, 140, and 250 bp upstream of the TSS. These peaks may correspond to the first nucleosome and the nucleosome-depleted region (NDR) upstream of the TSS (Ulz et al. 2016). In contrast to earlier reports that indicated increased coverage at TSSs for short fragments (Snyder et al. 2016), we observed a decrease in coverage near the TSS, possibly due to differences in purification methods. The coverage patterns indicated that high-expression genes had reduced coverage upstream of the TSS, while low-expression genes showed increased coverage. Recent studies have shown that ultra-short fragments tend to be hypomethylated at upstream transcription sites (Cheng et al. 2024), and differentially methylated ultra-short fragments exhibited distinct distribution patterns near the TSS. These findings suggest that the distribution of ultra-short fragments may be influenced by methylation status and other regulatory factors.

The enrichment of ultra-short fragment peaks in gene regulatory regions, such as promoter and 5’ UTR, underscores their potential role in gene regulation. We also observed increased coverage of these fragments at DHSs and ATAC-seq peaks, both markers of accessible chromatin. The significant decrease in coverage at methylated sites further supports the idea that ultra-short fragments reflect the chromatin state of blood and placental cells (Quina et al. 2006). Compared to placenta-specific DHSs and ATAC-seq peaks, increased coverage was observed in ultra-short cfDNA derived from pregnant women but not non-pregnancy women, suggesting a contribution from fetal open chromatin. Given that fetal DNA constitutes approximately 10% of total cfDNA, there was limited colocalization with placental peaks. However, the DHS and ATAC-seq peaks accounted for only a small fraction of the ultra-short cfDNA peaks, indicating that open chromatin may not be the primary source of these fragments. Transcription factor protection may also contribute to the formation of ultra-short fragments, but as our results and previous studies have shown, these peaks account for only a small proportion of the ultra-short peaks (Hisano et al. 2021). Further comparison between pregnancy and non-pregnancy revealed that most of the peaks were shared. Functional annotation of the pregnancy-enriched peaks showed significant involvement in the MAPK and PI3K-Akt signaling pathways, as well as processes related to immunity and development, highlighting their relevance to gene regulation during pregnancy.

C-rich sequences were prevalent in the ultra-short fragments, and potential G4 formations were identified (Hisano et al. 2021). G4 motifs play roles in cellular differentiation and development (Renčiuk et al. 2017; Zyner et al. 2022; David et al. 2016). Our results suggested that G4 motif formation may differ during pregnancy, warranting further investigation into the G4 signature in blood and placental cells. Moreover, G4 and methylation were found to be mutually exclusive at CpG islands. The decreased coverage at methylated sites could be explained by the fact that highly methylated CpG islands (CGIs) tend to be associated with unstable G4 structures (Jara-Espejo and Line 2020). Therefore, the relationship between G4 and methylation at CpG islands could be explored as a potential predictor of DNA methylation status during pregnancy.

Preeclampsia is a major pregnancy complication that poses risks to both mother and baby. Early prediction of preeclampsia could facilitate timely interventions, such as prophylactic treatments. Most cell-free nucleic acids in pregnant women are derived from maternal hematopoietic cells and placental trophoblasts (Lo et al. 1998; Tsang et al. 2017), which provide insight into placental cell dynamics. Previous studies have emphasized the importance of cfDNA and cfRNA in understanding placental function and early gene expression changes associated with preeclampsia (Guo et al. 2020; Moufarrej et al. 2022). Our findings on the characteristics of ultra-short cfDNA further suggest a link to abnormal gene expression modulation in this condition.

In this study, we identified distinct genomic regions associated with development, and these characteristics of ultra-short cfDNA may reflect abnormal gene expression modulation in pathological conditions. However, the precise mechanisms underlying these changes require further investigation. Using deep sequencing data, we identified differentially covered regions that contributed to a model with an AUC of 0.90 in the training set and 0.86 in the test set, demonstrating the diagnostic potential of ultra-short cfDNA for preeclampsia.

However, several limitations should be considered. Firstly, the sample size for both the training and test cohorts is relatively small, and future studies with larger, more diverse cohorts are needed to confirm the findings. Another limitation is the lack of stratified analysis for different clinical subtypes of preeclampsia. Different forms of preeclampsia may have distinct molecular signatures, and future studies should aim to investigate whether ultra-short cfDNA can be used to stratify patients based on their clinical characteristics. Moreover, the precise biological mechanisms underlying the generation of ultra-short cfDNA and its association with regulatory processes in pregnancy remain unclear. Future research should also explore the role of G4 motifs in pregnancy, as they may provide valuable insights into the molecular underpinnings of pregnancy complications. Finally, the potential utility of ultra-short cfDNA as a predictive marker for preeclampsia in earlier pregnancy stages should be evaluated in prospective studies, particularly those that include samples from asymptomatic women in the second trimester.

## Conclusion

In summary, our study comprehensively characterizes ultra-short cfDNA in maternal blood and highlights its preliminary potential as a non-invasive biomarker for the early prediction of preeclampsia. Future studies with larger cohorts and stratified clinical subtypes are needed to further validate these findings and explore the full clinical potential of ultra-short cfDNA as a diagnostic tool for pregnancy complications.

## Supplementary Information

Supplementary Material 1. Table S1: The differentially abundant peaks of ultra-short fragments of non-pregnant and pregnant women

Supplementary Material 2. Table S2: The differentially abundant ultra-short fragments peaks of preeclampsia patients and healthy controls

Supplementary Material 3. Table S3: Number of sequencing reads of the discovery cohort, the training cohort and the test cohort

Supplementary Material 4. Table S4: The CPM of the ultra-short fragments peaks used for model building in the training cohort and test cohort

Supplementary Material 5. Figure S1: The characteristics of different mapped fragments:: The length distribution of nuclear mapped fragments and mitochondrial mapped fragments.: The ratio of the mapping component of ultra-short fragments

Supplementary Material 6. Figure S2: The length distribution of cfDNA in plasma of pregnant and non-pregnant women

Supplementary Material 7. Figure S3: The enrichment of peaks called in sequencing data of plasma from pregnant and non-pregnant women

Supplementary Material 8. Figure S4: The ratio of nucleotides at the end of the ultra-short fragments. Boxplot of the ratio of the bases at the end

Supplementary Material 9. Figure S5: Heatmap of four nucleotides of the ends of the ultra-short fragments

Supplementary Material 10. Figure S6: Increased coverage of the ultra-short fragments on the open chromatin regions of blood cells.: Aggregation plots of read coverage of ultra-short fragments on the peaks of DHS of blood cells.: Aggregation plots of read coverage of ultra-short fragments on the peaks of ATAC of blood cells.: Aggregation plots of read coverage of ultra-short fragments on the methylation region of blood cells

Supplementary Material 11. Figure S7: Increased coverage of the ultra-short fragments on the open chromatin regions of the placenta.: Aggregation plots of read coverage of ultra-short fragments on the specific peaks of placental DHS.: Aggregation plots of read coverage of ultra-short fragments on the specific peaks of placental ATAC

Supplementary Material 12. Figure S8: Overlap of the peaks with cell type-specific transcription factor binding sites.: Overlap ratio of the peaks with the blood cell-specific transcription factors sites.: Overlap ratio of the peaks with the placental-specific transcription factor sites

Supplementary Material 13. Figure S9: Nucleobase frequency of the peaks of ultra-short fragments.: Density map showing base C of the peaks and the randomly selected 50-mers from the reference genome.: Density map of C skew of the peaks and the randomly chosen 50-mers from the reference genome.: Density map of base A, T, and G of the peaks and the randomly selected 50-mers from the reference genome, respectively

Supplementary Material 14. Figure S10: The diagram of workflow for feature selection and model building

Supplementary Material 15. Figure S11: Diagnostic potential of the relative coverage of long fragments at TSS for preeclampsia.: Heatmap for the selected TSS coverage in high-depth data.: AUC curve of the built SVM model for the training cohort.: AUC curve of the built SVM model for the test cohort

Supplementary Material 16. Code for Bioinformatics

## Data Availability

Sequence data supporting the results of this study have been deposited in the CNGB Sequence Archive under the primary accession number CNP0005305.

## Abbreviations

cfDNA: Cell-free DNA
cffDNA: Cell-free fetal DNA
ctDNA: circulating tumor DNA
NIPS: non-invasive prenatal screening
NGS: Next Generation Sequencing
SPRI: solid-phase reversible immobilization
G4: G-quadruplex
DHSs: DNase I hypersensitive sites
ATAC-seq: Assay for Transposase-Accessible Chromatin sequencing
TFBS: transcription factor binding sites
TSS: transcription start sites
CPM: counts per million
AUC: Area Under the Curve
NDR: nucleosome-depleted region
PE: Preeclampsia
