# Supplementary figures and images for: Characterization of Ultra-Short plasma Cell-Free DNA in maternal blood and its preliminary potential as a screening marker for preeclampsia
Source: Mol Med. 2025 Jul 12;31:256. doi: 10.1186/s10020-025-01307-1 (PMC12255149; doi:10.1186/s10020-025-01307-1)

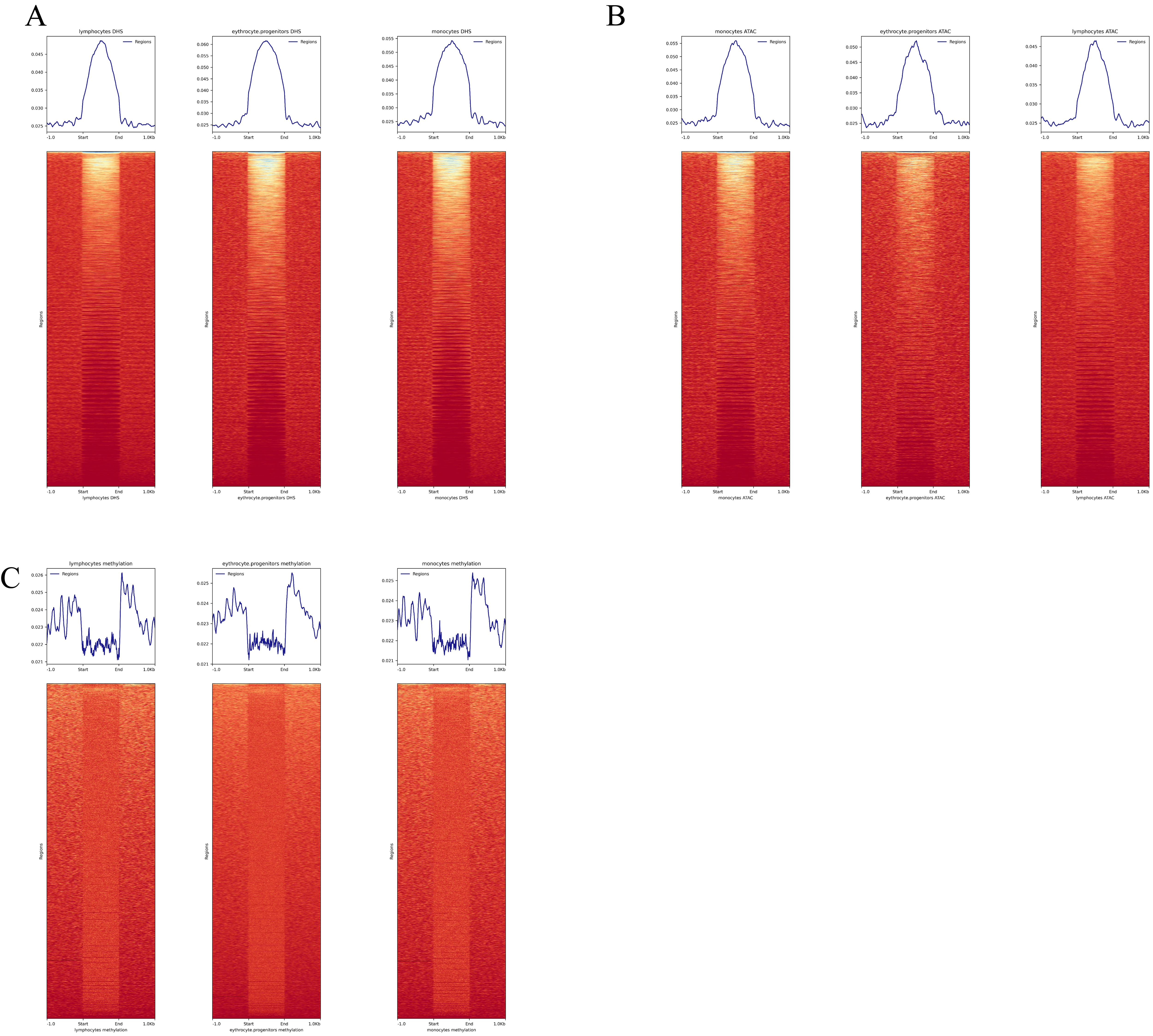

Supplement: Supplementary file 1 — Supplementary Material 1. Table S1: The differentially abundant peaks of ultra-short fragments of non-pregnant and pregnant women [file 10020_2025_1307_MOESM1_ESM.tif]

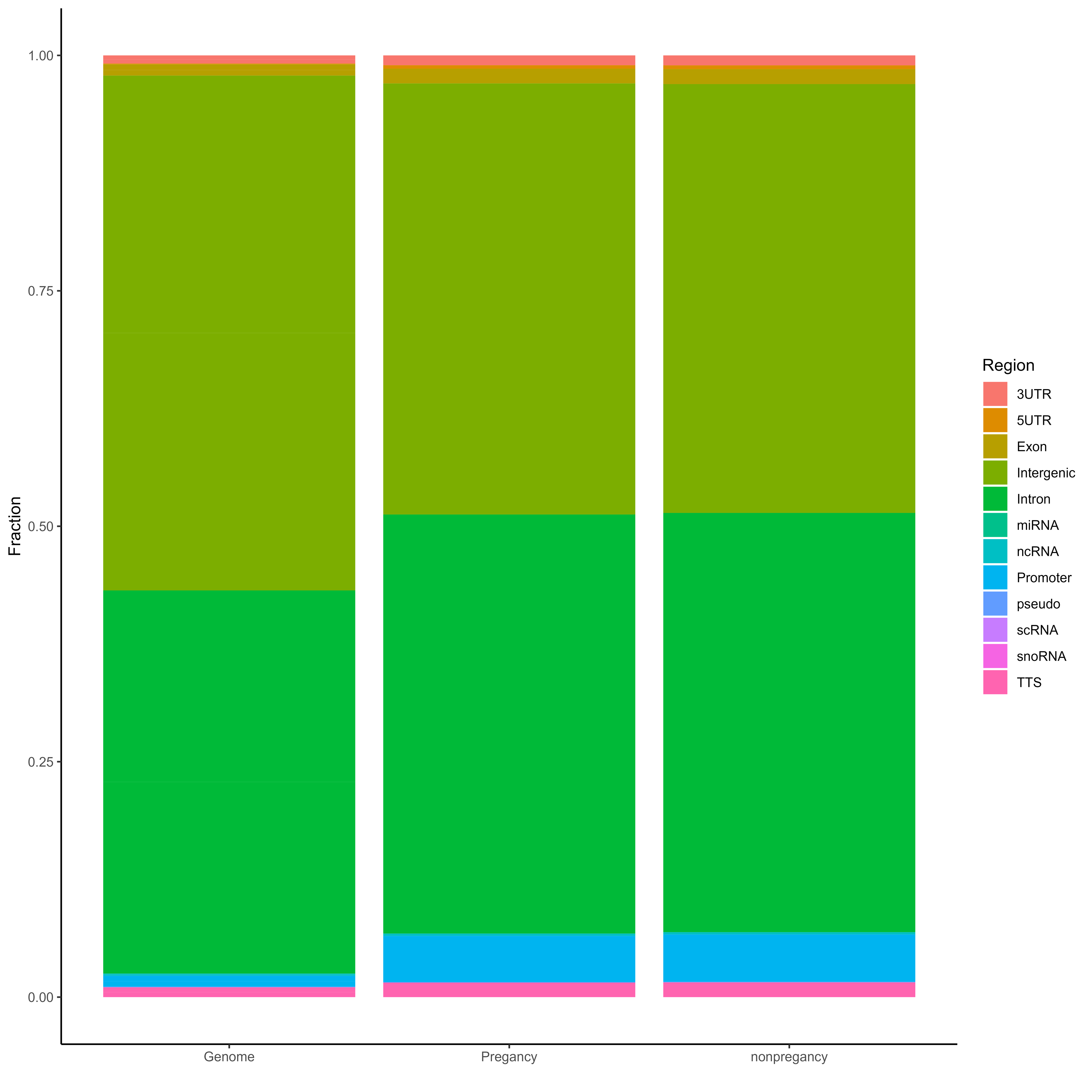

Supplement: Supplementary file 2 — Supplementary Material 2. Table S2: The differentially abundant ultra-short fragments peaks of preeclampsia patients and healthy controls [file 10020_2025_1307_MOESM2_ESM.tif]

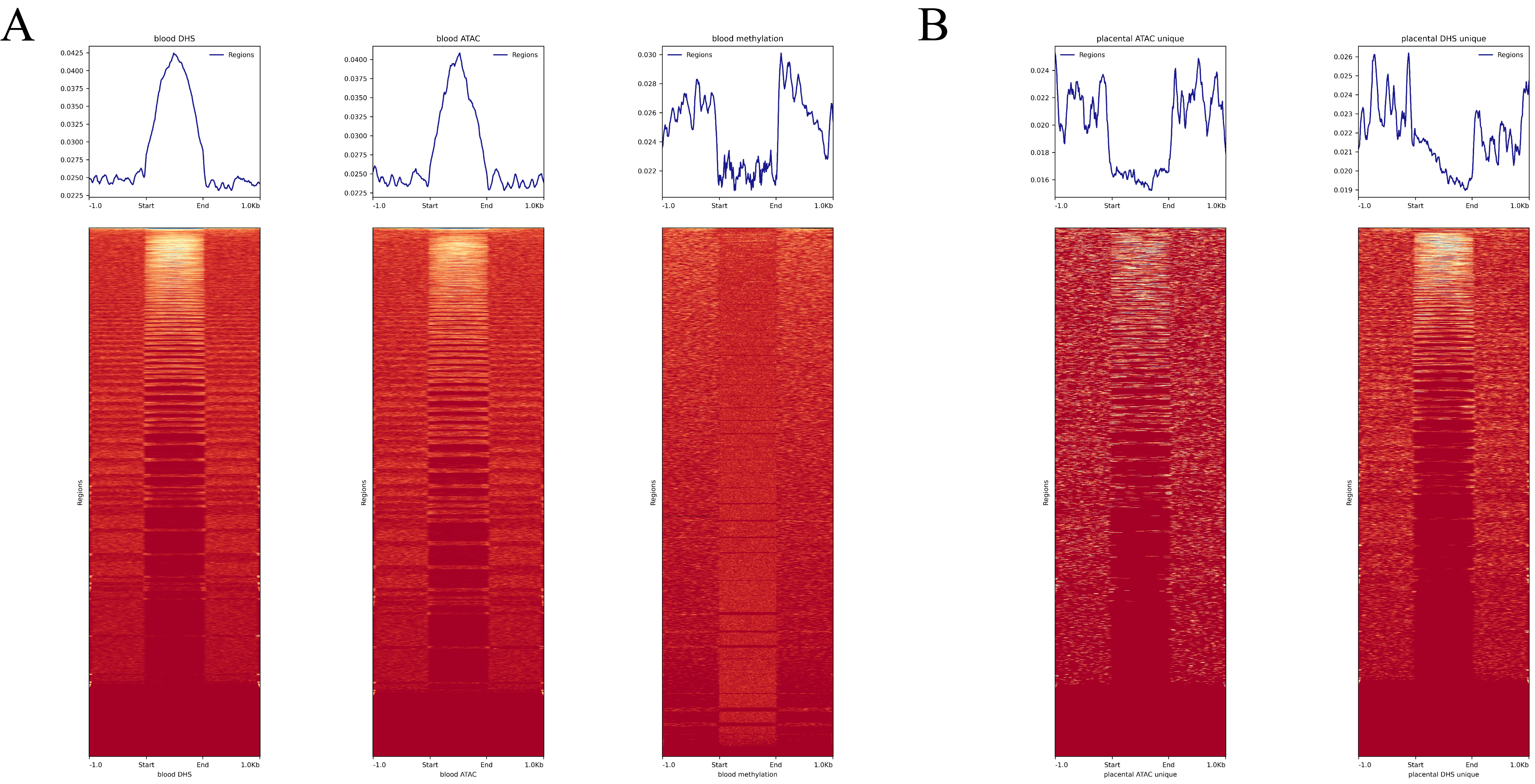

Supplement: Supplementary file 3 — Supplementary Material 3. Table S3: Number of sequencing reads of the discovery cohort, the training cohort and the test cohort [file 10020_2025_1307_MOESM3_ESM.tif]

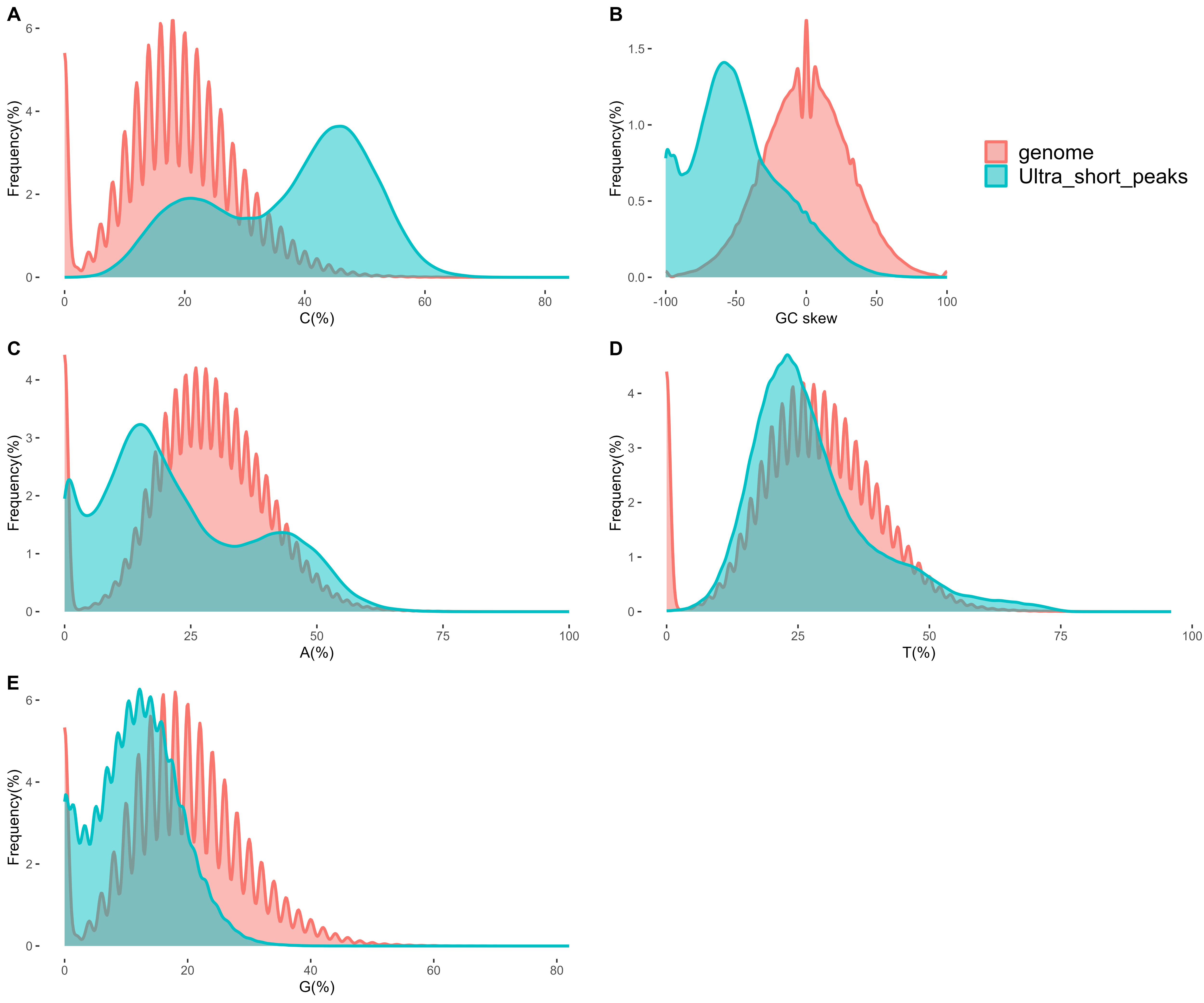

Supplement: Supplementary file 4 — Supplementary Material 4. Table S4: The CPM of the ultra-short fragments peaks used for model building in the training cohort and test cohort [file 10020_2025_1307_MOESM4_ESM.tif]

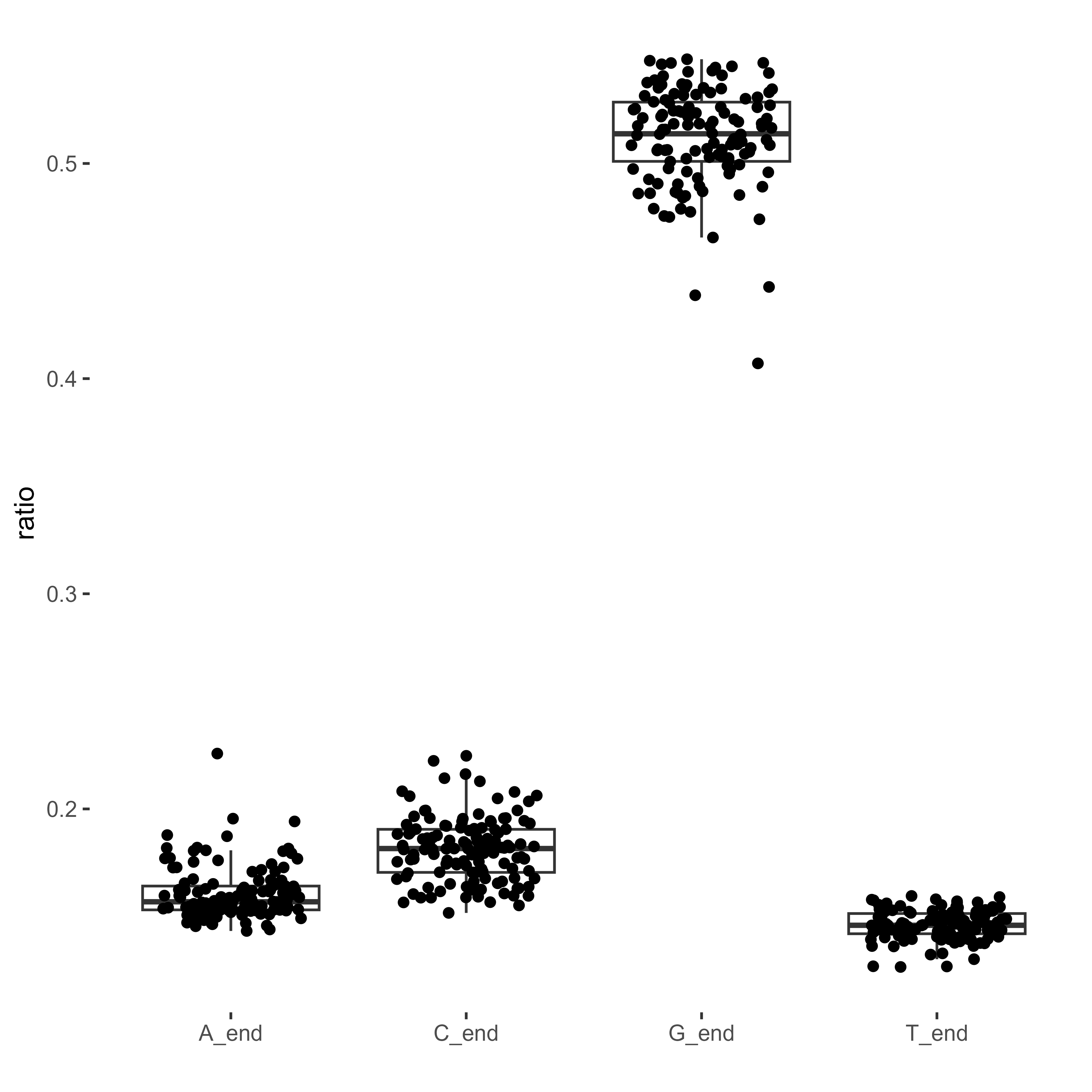

Supplement: Supplementary file 5 — Supplementary Material 5. Figure S1: The characteristics of different mapped fragments:: The length distribution of nuclear mapped fragments and mitochondrial mapped fragments.: The ratio of the mapping component of ultra-short fragments [file 10020_2025_1307_MOESM5_ESM.tif]

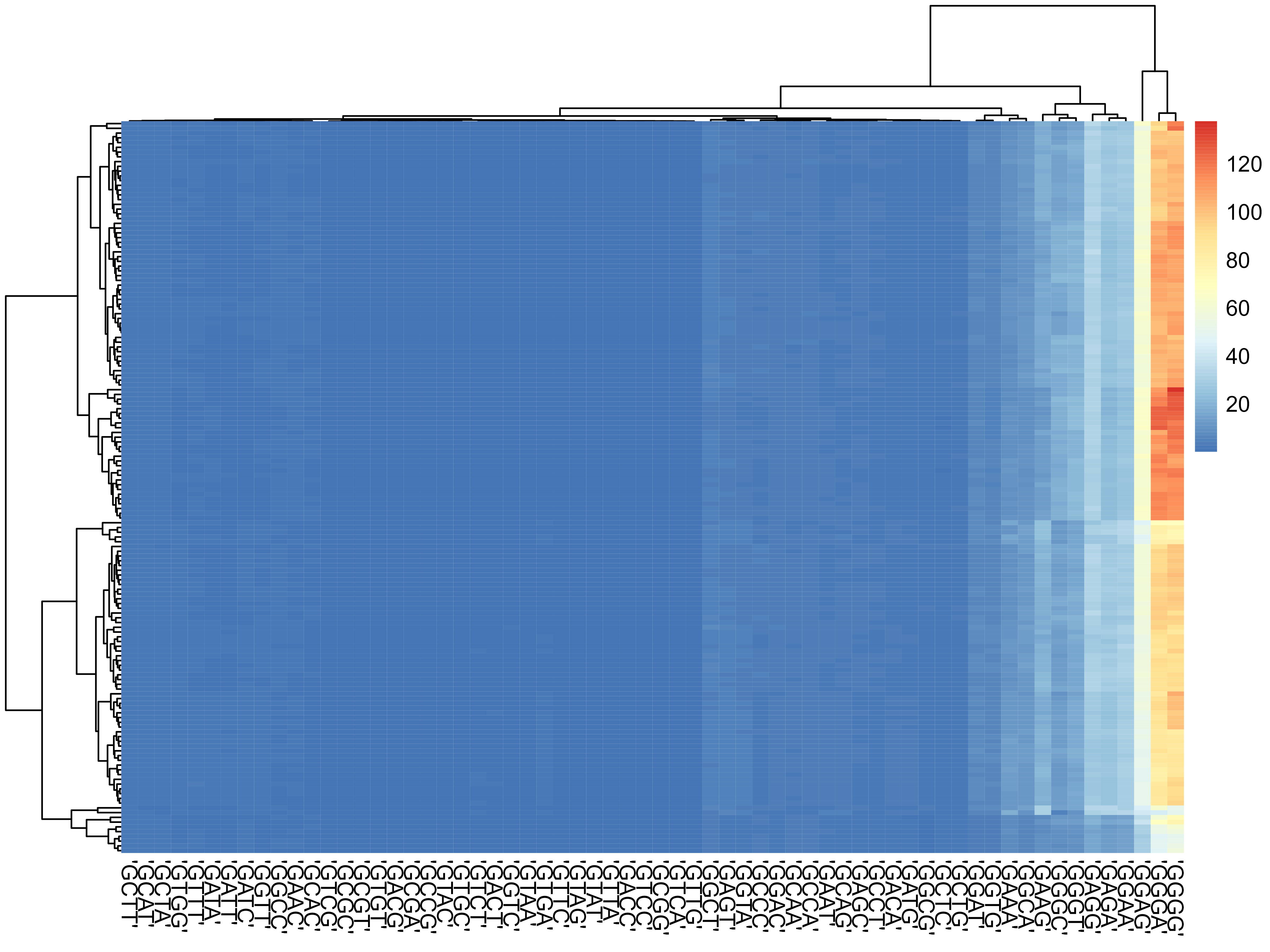

Supplement: Supplementary file 6 — Supplementary Material 6. Figure S2: The length distribution of cfDNA in plasma of pregnant and non-pregnant women [file 10020_2025_1307_MOESM6_ESM.tif]

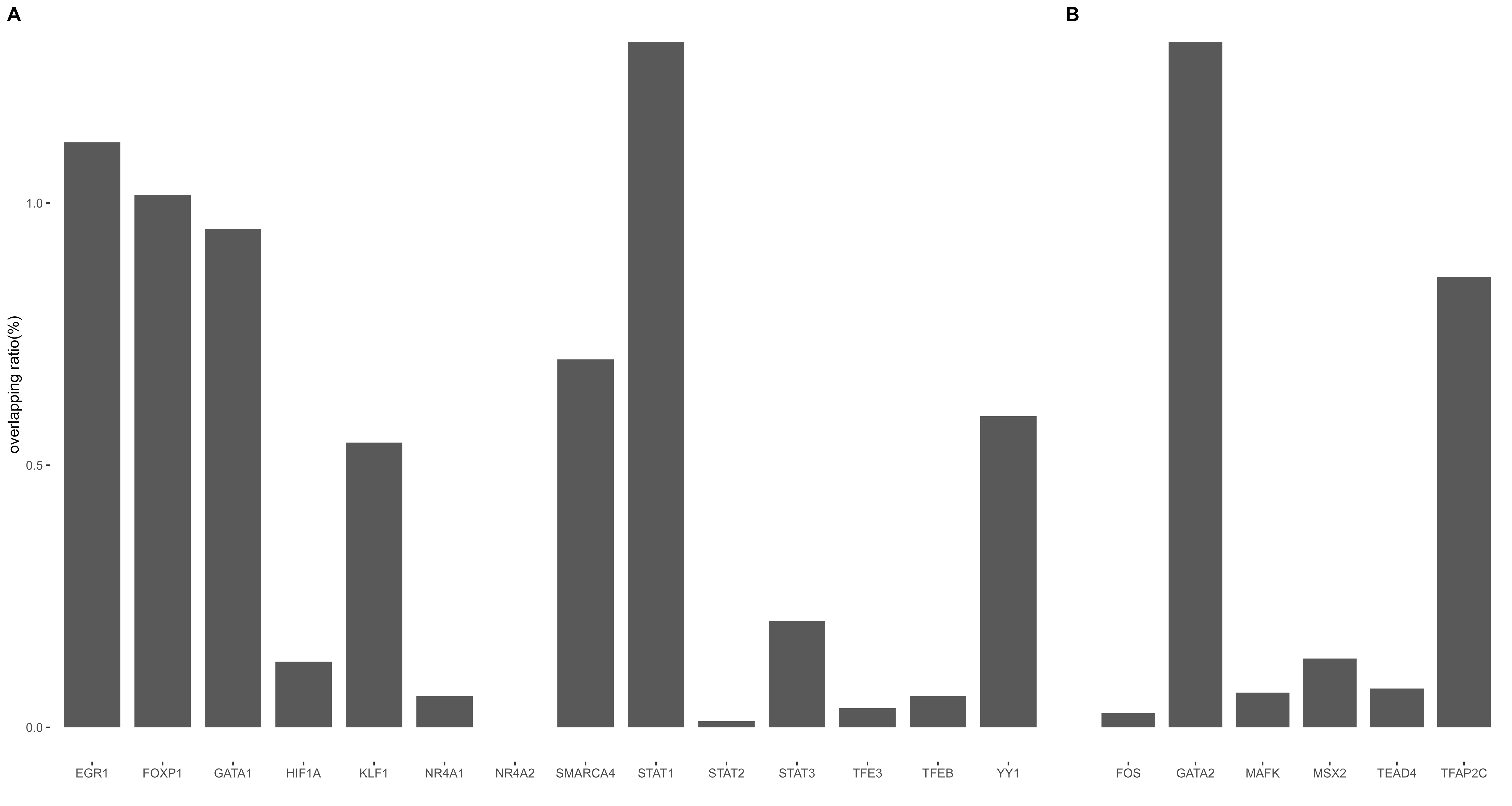

Supplement: Supplementary file 7 — Supplementary Material 7. Figure S3: The enrichment of peaks called in sequencing data of plasma from pregnant and non-pregnant women [file 10020_2025_1307_MOESM7_ESM.tif]

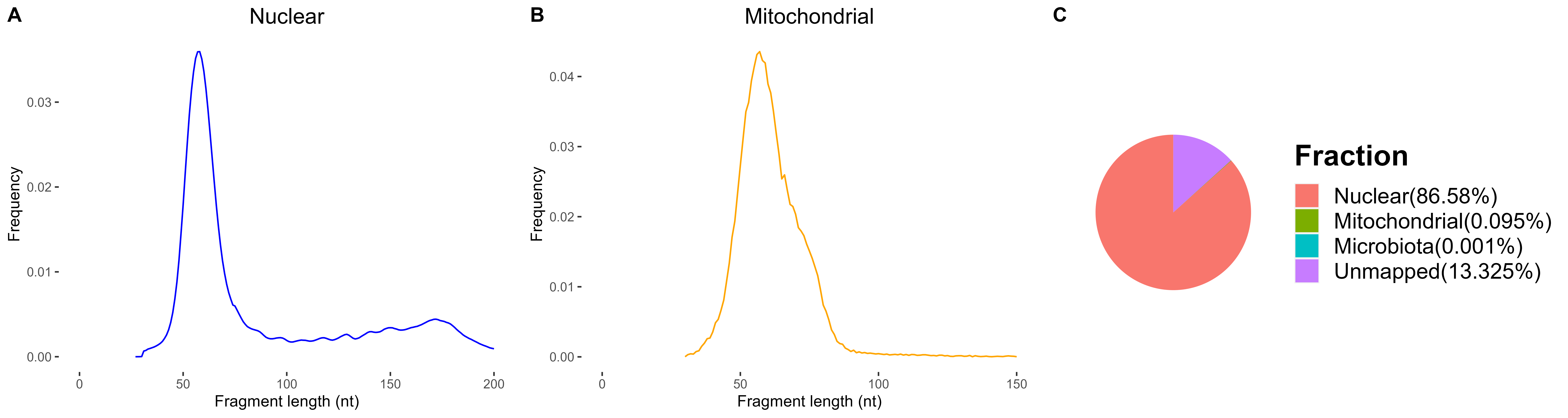

Supplement: Supplementary file 8 — Supplementary Material 8. Figure S4: The ratio of nucleotides at the end of the ultra-short fragments. Boxplot of the ratio of the bases at the end [file 10020_2025_1307_MOESM8_ESM.tif]

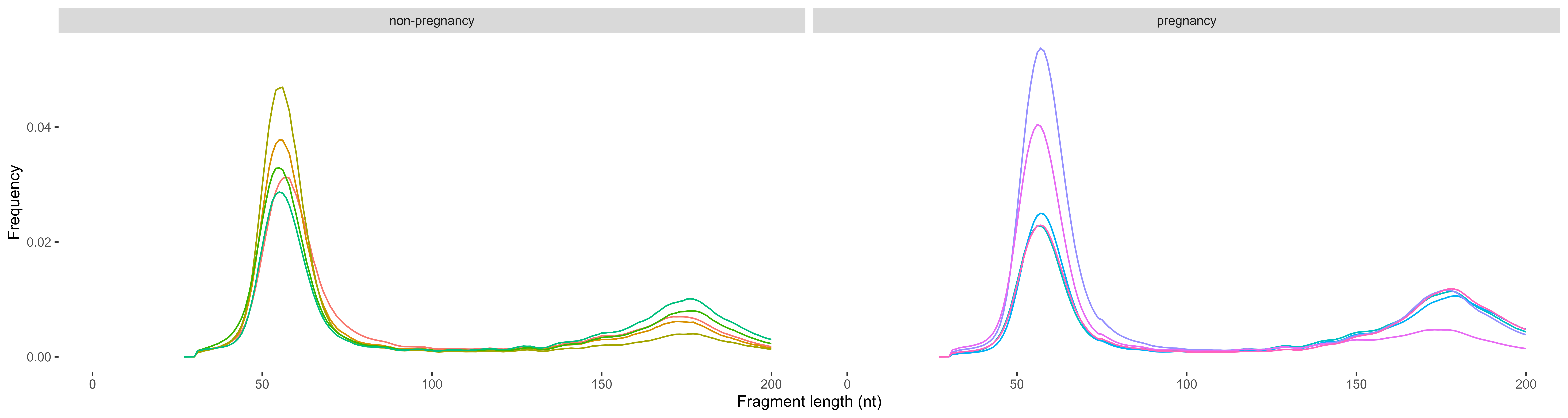

Supplement: Supplementary file 9 — Supplementary Material 9. Figure S5: Heatmap of four nucleotides of the ends of the ultra-short fragments [file 10020_2025_1307_MOESM9_ESM.tif]

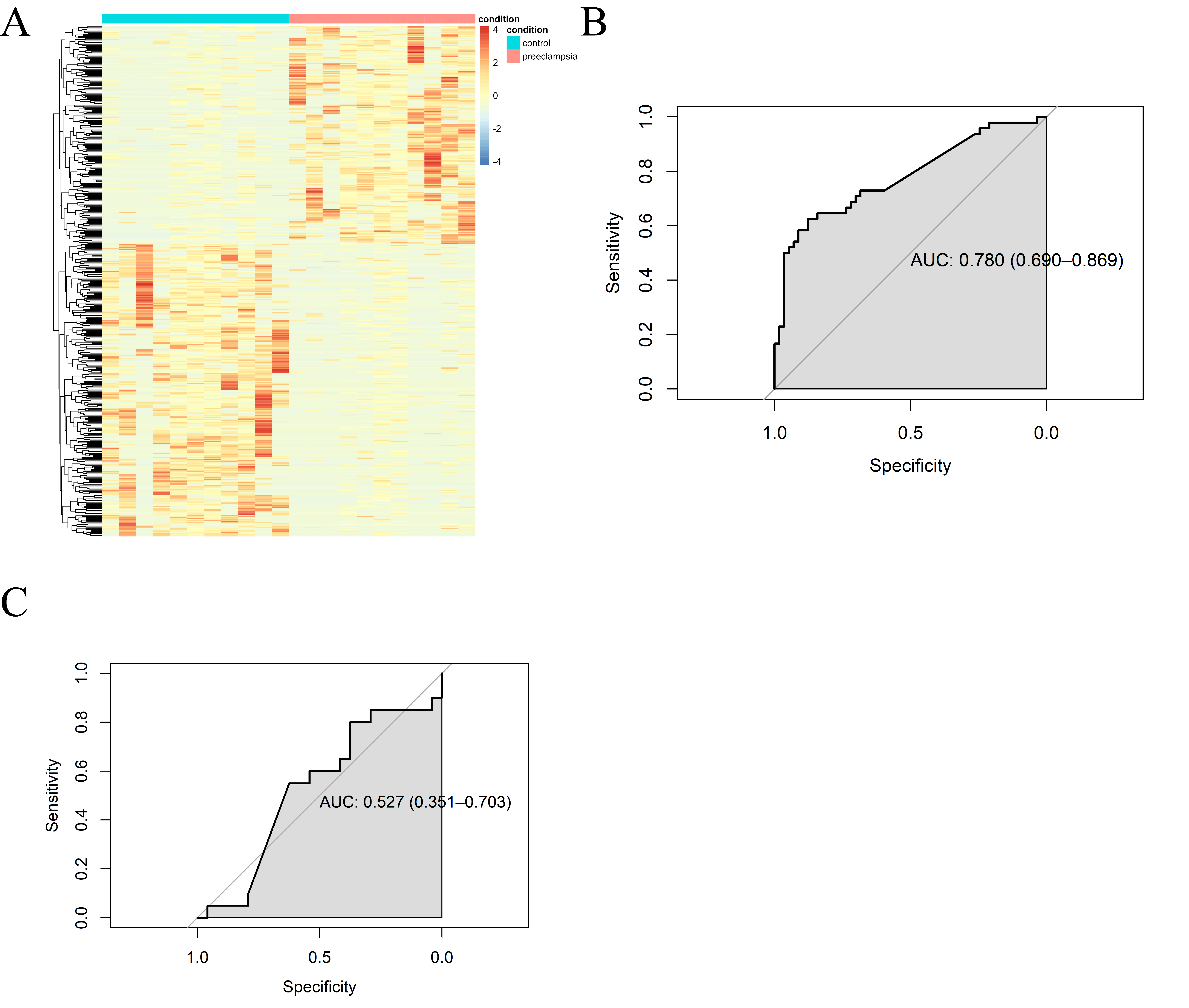

Supplement: Supplementary file 10 — Supplementary Material 10. Figure S6: Increased coverage of the ultra-short fragments on the open chromatin regions of blood cells.: Aggregation plots of read coverage of ultra-short fragments on the peaks of DHS of blood cells.: Aggregation plots of read coverage of ultra-short fragments on the peaks of ATAC of blood cells.: Aggregation plots of read coverage of ultra-short fragments on the methylation region of blood cells [file 10020_2025_1307_MOESM10_ESM.tif]

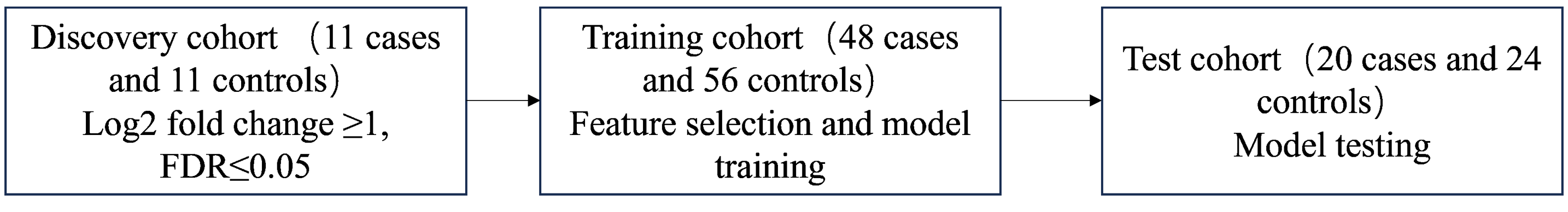

Supplement: Supplementary file 14 — Supplementary Material 14. Figure S10: The diagram of workflow for feature selection and model building [file 10020_2025_1307_MOESM14_ESM.tif]
