# Supplementary material for: Characterization of Ultra-Short plasma Cell-Free DNA in maternal blood and its preliminary potential as a screening marker for preeclampsia
Source: Mol Med. 2025 Jul 12;31:256. doi: 10.1186/s10020-025-01307-1 (PMC12255149; doi:10.1186/s10020-025-01307-1)
Supplement: Supplementary file 15 — Supplementary Material 15. Figure S11: Diagnostic potential of the relative coverage of long fragments at TSS for preeclampsia.: Heatmap for the selected TSS coverage in high-depth data.: AUC curve of the built SVM model for the training cohort.: AUC curve of the built SVM model for the test cohort [file 10020_2025_1307_MOESM15_ESM.docx]

**Supplementary file**

**Code for Bioinformatics**

**Trimming and mapping**

**FASTQ**

**filtered and trimming**

Fastp:Remove low-quality reads

$fastp (no option)

**Mapping**

$bowtie2 -q -p 10 -x hg19

**BAM, uniquely mapped reads**

$samtools view -b -F 4 -q 1 input.bam > output.bam

$java -jar "picard.jar" MarkDuplicates REMOVE_DUPLICATES=true I=output.bam O=rmdup.txt

**Separate of mapped reads and peak calling**

**BAM, extracted by fragment size**

$awk '/^@/ || ($9 >= -70 && $9 <= 70)' | awk '/^@/ || ($9 <= -30 || $9 >= 30)' > 30_70.sam

$awk '/^@/ || ($9 >= -150 && $9 <= 150)' | awk '/^@/ || ($9 <= -100 || $9 >= 100)' > 100_150.sam

$awk '/^@/ || ($9 >= -1000 && $9 <= 1000)' | awk '/^@/ || ($9 < -150 || $9 > 150)' > 1000.sam

**BAM, separated with mapped strand**

$samtools view -h -f 128 -F 16 30_70.sam >forward.sam

$samtools view -f 80 30_70.sam >>forward.sam

$samtools view -h -f 64 -F 16 30_70.sam >.reverse.sam

$samtools view -f 144 30_70.sam >>reverse.sam

**MACS2, peak calling**

$macs2 callpeak -t forward.sorted.bam -f BAMPE -g hs -n sample_forward --outdir outdir

$macs2 callpeak -t reverse.sorted.bam -f BAMPE -g hs -n sample_reverse --outdir outdir

**Calculation of the read coverage**

**BAMCOVERAGE, convert bam to bigwig**

$bamCoverage -p 10 --binSize 10 --normalizeUsing BPM --exactScaling --bam forward.bam -o forward.bw

$bamCoverage -p 10 --binSize 10 --normalizeUsing BPM --exactScaling --bam reverse.bam -o reverse.bw

**Peak annotation**

annotatePeaks.pl peak.bed hg19 –annStats > peak_ann.xls

**get the fasta of the peakfile**

bedtools getfasta -fi hg19.fasta -bed peaks.bed > peaks.fasta

**get the statistics of the the previous fastafile**

seqkit fx2tab peaks.fasta -g -G -B A -B T -B C -B G >peakstas.xls

**get the random fragment of genome**

bedtools random -g /data10/luqin/ultra_short/gene_region_bed/genome_size.txt -l 50 -n 100000

**draw the heatmap**

plotHeatmap -m  bed.matrix.gz -o bed.png --sortUsing mean --averageType mean --startLabel Start --endLabel End --perGroup --samplesLabel  --regionsLabel

**blood DNS seq peaks**

cat DNS.Bld.50.AllAg.CD34PULUS.bed DNS.Bld.50.AllAg.CD34PULUS_cells.bed DNS.Bld.50.AllAg.Erythroid_Cells.bed DNS.Bld.50.AllAg.Hematopoietic_Stem_Cells.bed DNS.Bld.50.AllAg.Peripheral_blood_stem_cells.bed | sortBed | mergeBed > eythrocyte.progenitors_DHS_hg19.bed

cat DNS.Bld.50.AllAg.B-cell_BRACKETLCD19PULUSBRACKETR.bed DNS.Bld.50.AllAg.B_cells.bed DNS.Bld.50.AllAg.CD20PULUS_B_cells.bed DNS.Bld.50.AllAg.CD20PULUS.bed DNS.Bld.50.AllAg.CD3PULUS.bed DNS.Bld.50.AllAg.CD4PULUS_T_cells.bed DNS.Bld.50.AllAg.CD4PULUS.bed DNS.Bld.50.AllAg.CD4PULUS_Th1.bed DNS.Bld.50.AllAg.CD56PULUS.bed DNS.Bld.50.AllAg.CD8PULUS.bed DNS.Bld.50.AllAg.PBMC.bed DNS.Bld.50.AllAg.Th17_Cells.bed DNS.Bld.50.AllAg.Th1_Cells.bed DNS.Bld.50.AllAg.Th2_Cells.bed DNS.Bld.50.AllAg.Treg.bed DNS.Bld.50.AllAg.Treg_Wb83319432.bed | sortBed | mergeBed > lymphocytes_DHS_hg19.bed

cat DNS.Bld.50.AllAg.Dendritic_Cells.bed DNS.Bld.50.AllAg.Macrophages.bed DNS.Bld.50.AllAg.Monocytes.bed DNS.Bld.50.AllAg.Monocytes-CD14PULUS.bed | sortBed | mergeBed > monocytes_DHS_hg19.bed

**placenta DNS peaks**

cat DNS.Plc.50.AllAg.Placenta.bed DNS.Plc.50.AllAg.Trophoblasts.bed | sortBed | mergeBed > placenta_DHS_hg19.bed

**blood ATAC**

cat ATC.Bld.50.AllAg.CD34PULUS.bed ATC.Bld.50.AllAg.Erythroid_progenitors.bed ATC.Bld.50.AllAg.Erythroid_Cells.bed ATC.Bld.50.AllAg.Hematopoietic_Stem_Cells.bed | sortBed | mergeBed > eythrocyte.progenitors_ATAC_hg19.bed

cat ATC.Bld.50.AllAg.B-cell_BRACKETLCD19PULUSBRACKETR.bed ATC.Bld.50.AllAg.B_cells.bed  ATC.Bld.50.AllAg.CD34PULUS.bed  ATC.Bld.50.AllAg.CD4-Positive_T-Lymphocytes.bed ATC.Bld.50.AllAg.CD4PULUS_T_cells.bed ATC.Bld.50.AllAg.CD8PULUS_T_cells.bed ATC.Bld.50.AllAg.CD8-Positive_T-Lymphocytes.bed ATC.Bld.50.AllAg.PBMC.bed ATC.Bld.50.AllAg.Th17_Cells.bed ATC.Bld.50.AllAg.Th1_Cells.bed ATC.Bld.50.AllAg.Th2_Cells.bed ATC.Bld.50.AllAg.Treg.bed | sortBed | mergeBed > lymphocytes_ATAC_hg19.bed

cat ATC.Bld.50.AllAg.Dendritic_Cells.bed ATC.Bld.50.AllAg.Macrophages.bed ATC.Bld.50.AllAg.Monocytes.bed ATC.Bld.50.AllAg.Monocytes-CD14PULUS.bed | sortBed | mergeBed > monocytes_ATAC_hg19.bed

**white cell ATAC**

cat ATC.Bld.50.AllAg.Neutrophils.bed | sortBed | mergeBed > white_cell_ATAC_hg19.bed

**placenta ATAC**

cat ATC.Plc.50.AllAg.Syncytiotrophoblast.bed  ATC.Plc.50.AllAg.Trophoblasts.bed | sortBed | mergeBed > placetal_ATAC_hg19.bed

**blood methylation**

cat BSF.Bld.bs.AllAg.CD34PULUS.bed BSF.Bld.bs.AllAg.Erythroid_progenitors.bed BSF.Bld.bs.AllAg.Hematopoietic_Stem_Cells.bed | sortBed | mergeBed > eythrocyte.progenitors_methy_hg19.bed

cat BSF.Bld.bs.AllAg.B-cell_BRACKETLCD19PULUSBRACKETR.bed BSF.Bld.bs.AllAg.B-Lymphocytes.bed BSF.Bld.bs.AllAg.CD3PULUS_cells.bed BSF.Bld.bs.AllAg.CD4PULUS.bed BSF.Bld.bs.AllAg.CD4-Positive_T-Lymphocytes.bed BSF.Bld.bs.AllAg.CD56PULUS_cells BSF.Bld.bs.AllAg.CD8PULUS.bed BSF.Bld.bs.AllAg.PBMC.bed | sortBed | mergeBed > lymphocytes_methy_hg19.bed

cat BSF.Bld.bs.AllAg.Dendritic_Cells.bed BSF.Bld.bs.AllAg.Macrophages.bed BSF.Bld.bs.AllAg.Monocytes.bed BSF.Bld.bs.AllAg.Monocytes-CD14PULUS.bed | sortBed | mergeBed > monocytes_methy_hg19.bed

**white cell methylation**

cat BSF.Bld.bs.AllAg.Neutrophils.bed BSF.Bld.bs.AllAg.White_blood_cells.bed | sortBed | mergeBed > white_cell_methy_hg19.bed

**placenta methylation**

cat BSF.Plc.bs.AllAg.Chorinic_villus.bed BSF.Plc.bs.AllAg.Cytotrophoblast.bed BSF.Plc.bs.AllAg.Extravillous_trophoblast.bed BSF.Plc.bs.AllAg.Placenta.bed BSF.Plc.bs.AllAg.Placenta_villi.bed BSF.Plc.bs.AllAg.Trophoblasts.bed | sortBed | mergeBed > placenta_methy_hg19.bed

cat data_Human_20blood_20monocytes_20derived.txt data_Blood_20monocytes.txt data_Monocytes-CD14_2B_RO01746.txt data_Monocyte-derived_20macrophage.txt data_monocyte_20derived_20macrophages_20_MDM.txt | sortBed | mergeBed > monocytes_CTCF_hg19.bed

cat data_Peripheral_20blood_20neutrophils.txt | sortBed | mergeBed > white_cell_CTCF_hg19.bed

**merge blood DHS**

cat eythrocyte.progenitors_DHS_hg19.bed lymphocytes_DHS_hg19.bed monocytes_DHS_hg19.bed | sortBed | mergeBed > blood_DHS_hg19.bed

**placenta unique DHS**

bedtools subtract -A -a placenta_DHS_hg19.bed -b blood_DHS_hg19.bed > placenta_DHS_unique_hg19.bed

**merge blood ATAC**

cat eythrocyte.progenitors_ATAC_hg19.bed lymphocytes_ATAC_hg19.bed monocytes_ATAC_hg19.bed white_cell_ATAC_hg19.bed | sortBed | mergeBed > blood_ATAC_hg19.bed

**placenta unique DHS**

bedtools subtract -A -a placetal_ATAC_hg19.bed -b blood_ATAC_hg19.bed > placenta_ATAC_unique_hg19.bed

**merge blood methylation**

cat eythrocyte.progenitors_methy_hg19.bed lymphocytes_methy_hg19.bed monocytes_methy_hg19.bed white_cell_methy_hg19.bed | sortBed | mergeBed > blood_methy_hg19.bed

**placenta unique methylation**

bedtools subtract -A -a placenta_methy_hg19.bed -b blood_methy_hg19.bed > placenta_methy_unique_hg19.bed

**transcriptional factor binding sites of blood or placenta from chipatlas**

**transcriptional factor for blood:** Oth.Bld.50.EGR1.AllCell.bed,Oth.Bld.50.FOXP1.AllCell.bed,Oth.Bld.50.GATA1.AllCell.bed,Oth.Bld.50.HIF1A.AllCell.bed,Oth.Bld.50.KLF1.AllCell.bed,Oth.Bld.50.NR4A1.AllCell.bed,Oth.Bld.50.NR4A2.AllCell.bed,Oth.Bld.50.SMARCA4.AllCell.bed,Oth.Bld.50.STAT1.AllCell.bed,Oth.Bld.50.STAT2.AllCell.bed,Oth.Bld.50.STAT3.AllCell.bed,Oth.Bld.50.TFE3.AllCell.bed,Oth.Bld.50.TFEB.AllCell.bed,Oth.Bld.50.YY1.AllCell.bed

**transcriptional factor for placenta** :

Oth.Plc.50.FOS.AllCell.bed,Oth.Plc.50.GATA2.AllCell.bed,Oth.Plc.50.MAFK.AllCell.bed,Oth.Plc.50.MSX2.AllCell.bed,Oth.Plc.50.TEAD4.AllCell.bed,Oth.Plc.50.TFAP2C.AllCell.bed

bedtools bedfile sortBed | mergeBed > sorted_merged.bed

**download data from GSE63874**

**get the peaks from G4 seq data**

cat GSE63874_Na_K_PDS_plus_hits_intersect.bed  | sortBed | mergeBed > G4_seq_plus.bed

cat GSE63874_Na_K_PDS_minus_hits_intersect.bed | sortBed | mergeBed > G4_seq_minus.bed

**predicted G4 motif**

# fastaRegexFinder from https://github.com/dariober/bioinformatics-cafe/blob/master/fastaRegexFinder/fastaRegexFinder.py?raw=true

fastaRegexFinder.py --quiet -r '([gG]{3,}\w{1,7}){3,}[gG]{3,}' -f genome.fa > PQS_N1-7_hg19.bed

**Model building, r script**

library(caret)

library(pROC)

library(Boruta)

patition<-createDataPartition(group_usf$preeclampsia,p=0.7)

train_control<-trainControl(method = 'LOOCV',classProbs = TRUE, savePredictions = T)

feature_boruta<-Boruta(x= usf_data[patition$Resample1,],y = as.factor(group_usf$preelampsia[patition$Resample1,]),pValue=0.01, mcAdj=T,maxRuns=1000)

usf_data <- usf_data [,gsub('\\.','-',names(feature_boruta $finalDecision)[ feature_boruta$finalDecision=='Confirmed'])]

usf_model<-train(x=usf_data[patition$Resample1,],y= = as.factor(group_usf$preeclampsia[patition$Resample1]),method = 'svmRadial',trControl = train_control)

test_predict<-predict(usf_model,usf_data[-patition$Resample1,],type = 'prob')

train_predict<-predict(usf_model,usf_data[patition$Resample1,],type = 'prob')

roc_70_test<-roc(response=group_usf$preeclampsia[-patition$Resample1], test_predict$N,ci=T)

roc_70_train<-roc(response=group_usf$preeclampsia[patition$Resample1], train_predict$N,ci=T)
