# Supplementary material for: Characterization of Ultra-Short plasma Cell-Free DNA in maternal blood and its preliminary potential as a screening marker for preeclampsia
Source: Mol Med. 2025 Jul 12;31:256. doi: 10.1186/s10020-025-01307-1 (PMC12255149; doi:10.1186/s10020-025-01307-1)
Supplement: Supplementary file 16 — Supplementary Material 16. Code for Bioinformatics [file 10020_2025_1307_MOESM16_ESM.docx]

| Discovery cohort | | | |
| --- | --- | --- | --- |
|  | Control (n=11) | Preeclampsia (n=11) | Total (n=22) |
| Raw reads | 332.40±28.09(299.50,383.36) M | 355.23±16.98(322.20,386.41) M | 343.82±25.49(299.50,386.41) M |
| Clean reads | 321.56±29.31(291.59,379.12) M | 344.36±15.14(318.14,374.86) M | 332.96±25.58(291.59,379.12) M |
| Q20 | 0.95±0.03(0.91,0.97) | 0.94±0.04(0.88,0.97) | 0.95± 0.03( 0.88, 0.97) |
| Q30 | 0.90±0.06(0.81,0.94) | 0.89±0.07(0.78,0.94) | 0.90± 0.06( 0.78, 0.94) |
| Duplication | 0.37±0.10(0.25,0.50) | 0.35±0.10(0.13,0.50) | 0.36± 0.10( 0.13, 0.50) |
| Unique reads | 209.14±53.89(154.15,336.47) M | 255.86±85.53(178.32,479.41) M | 232.50±73.74(154.15,479.41) M |
| 30-70 bp fragment ratio | 0.60±0.15(0.40,0.82) | 0.65±0.10(0.49,0.79) | 0.62± 0.13( 0.40, 0.82) |
| Training and test cohort | | | |
|  | Control (n=80) | Preeclampsia (n=68) | Total (n=148) |
| Raw reads | 118.30±37.18(70.62,220.72) M | 127.13±18.49(91.24,173.10) M | 122.36±30.30(70.62,220.72) M |
| Clean reads | 116.66±36.66(70.05,217.82) M | 125.26±18.30(89.66,169.12) M | 120.61±29.89(70.05,217.82) M |
| Q20 | 0.94±0.01(0.92,0.96) | 0.95±0.01(0.93,0.96) | 0.94± 0.01( 0.92, 0.96) |
| Q30 | 0.85±0.01(0.82,0.89) | 0.86±0.02(0.83,0.89) | 0.86± 0.02( 0.82, 0.89) |
| Duplication | 0.14±0.06(0.03,0.30) | 0.11±0.04(0.03,0.22) | 0.12± 0.05( 0.03, 0.30) |
| Unique reads | 103.69±30.35(63.75,187.33) M | 107.93±15.58(71.17,148.83) M | 105.63±24.70 (63.75, 187.33) M |
| 30-70 bp fragment ratio | 0.65±0.12(0.40,0.96) | 0.63±0.14(0.25,0.87) | 0.64± 0.13( 0.25, 0.96) |

Supplementary Table 3

Number of sequencing reads of the discovery cohort, the training cohort and the test cohort

*M: million
